# How does carbon dioxide permeate cell membranes? A discussion of concepts, results and methods

**DOI:** 10.3389/fphys.2013.00382

**Published:** 2014-01-08

**Authors:** Volker Endeward, Samer Al-Samir, Fabian Itel, Gerolf Gros

**Affiliations:** ^1^Zentrum Physiologie, Vegetative Physiologie 4220, Medizinische Hochschule HannoverHannover, Germany; ^2^Departement Chemie, Universität BaselBasel, Switzerland

**Keywords:** carbon dioxide, membrane permeability, gas channels, membrane cholesterol, gas permeation measurements

## Abstract

We review briefly how the thinking about the permeation of gases, especially CO_2_, across cell and artificial lipid membranes has evolved during the last 100 years. We then describe how the recent finding of a drastic effect of cholesterol on CO_2_ permeability of both biological and artificial membranes fundamentally alters the long-standing idea that CO_2_—as well as other gases—permeates all membranes with great ease. This requires revision of the widely accepted paradigm that membranes never offer a serious diffusion resistance to CO_2_ or other gases. Earlier observations of “CO_2_-impermeable membranes” can now be explained by the high cholesterol content of some membranes. Thus, cholesterol is a membrane component that nature can use to adapt membrane CO_2_ permeability to the functional needs of the cell. Since cholesterol serves many other cellular functions, it cannot be reduced indefinitely. We show, however, that cells that possess a high metabolic rate and/or a high rate of O_2_ and CO_2_ exchange, do require very high CO_2_ permeabilities that may not be achievable merely by reduction of membrane cholesterol. The article then discusses the alternative possibility of raising the CO_2_ permeability of a membrane by incorporating protein CO_2_ channels. The highly controversial issue of gas and CO_2_ channels is systematically and critically reviewed. It is concluded that a majority of the results considered to be reliable, is in favor of the concept of existence and functional relevance of protein gas channels. The effect of intracellular carbonic anhydrase, which has recently been proposed as an alternative mechanism to a membrane CO_2_ channel, is analysed quantitatively and the idea considered untenable. After a brief review of the knowledge on permeation of O_2_ and NO through membranes, we present a summary of the ^18^O method used to measure the CO_2_ permeability of membranes and discuss quantitatively critical questions that may be addressed to this method.

## The classical concept: lipid bilayers and cell membranes are extremely permeable to gases

It has been a long-standing assumption in biology that all cell membranes let gases pass extremely easily by virtue of the high gas solubility of lipophilic gases in the membranes' lipid phase. This view was formulated as a general principle of the permeation of substances across cell membranes as early as between 1895 and 1901 by Ernest Overton (1895, 1896, 1899, 1901; see also review by Kleinzeller, 1997). Twenty years later, this same principle was shown to specifically hold for the gases CO_2_ and NH_3_ by Jacobs (1920a,b,^1922^). The idea seemed to be supported by measurements of the gas permeabilities of artificial phospholipid membranes, which for O_2_, CO_2_, and N_2_ as well as other gases, turned out to be extremely high, i.e., considerably higher than the permeabilities of small uncharged polar molecules such as water, urea, and glycerol, not to speak of larger, uncharged polar molecules such as sugars or even of ions. This view has entered most textbooks as a well-established fact valid also for cell membranes (Alberts et al., 2002). In the case of CO_2_, which is highly lipophilic, but almost equally hydrophilic at the same time, the experimentally determined CO_2_ permeability values (*P*_CO_2__) of lipid bilayers are around 1 cm/s (Gutknecht et al., 1977; Missner et al., 2008). How high this value is, may be appreciated by comparing it with the Cl^−^—HCO^−^_3_ permeability of the red cell membrane, which with a value of 1·10^−4^–1·10^−3^ cm/s is 3–4 orders of magnitude lower, although it is exceptionally high in comparison to other ion permeabilities.

The concept of Overton has later been extended to the “solubility-diffusion mechanism” by Finkelstein (1976). With this modification, Finkelstein took into account that membrane permeability may not only depend on the solubility in the membrane of the substance considered, but also on the diffusion resistance the substance encounters within the membrane.

The transport rate of a substance across a membrane, *dm*/*dt*, can be expressed by Fick's first diffusion law:

(1) $$dm/dt=-D_{\text{app}}\cdot A\cdot \Delta c_{w}/d,\text{ or}$$

(2) $$dm/dt=-P\cdot A\cdot \Delta c_{w}$$

with *D*_app_ representing the apparent membrane diffusion coefficient, *A* the diffusion area of the membrane, *d* the thickness of the membrane, *P* the membrane permeability, and Δ*c*_*w*_ the difference of concentrations in the water phase immediately adjacent to two sides of the membrane. If the true diffusivity of the substance within the membrane, *D*_*M*_, is to be considered, the concentration difference present within the membrane has to be expressed using the lipid/water partition coefficient *K*_*P*_:

(3) $$dm/dt=-D_{M}\cdot A\cdot \Delta c_{M}/d=-D_{M}\cdot A\cdot \Delta c_{w}\cdot K_{P}/d,$$

and, from Equations (2) and (3):

(4) $$P=D_{M}\cdot K_{P}/d.$$

Thus, according to Finkelstein's concept, the membrane permeability will vary with the lipid-water partition coefficient, but will in addition depend on intramembrane diffusivity.

While the intramembrane diffusion coefficients of gases have not been determined directly, the lipid-water partition coefficients are fairly well known. This is shown for several gases of biological interest in Table 1. Firstly, it is apparent that the gases O_2_, NO, N_2_, and CO behave quite similar in terms of water and lipid solubilities as well as partition coefficients. CO_2_, on the other hand, differs greatly from all these gases with respect to solubility in water and in lipid. CO_2_ exhibits a 20–50 times higher solubility in water than the other four gases, and in oil an about 10–20 times higher solubility. This results in partition coefficients that are only moderately smaller for CO_2_ than for the other gases: at most by a factor of 3–4 for the oil/water as well as for the phospholipid/water system. It is interesting to note that the gas partition coefficients in Table 1 in general appear quite similar for the oil/water and the phospholipid/water system. The last line of the table gives the measured *P*_CO_2__ in phospholipid membranes as mentioned above, together with the permeabilities predicted from *P*_CO_2__ by assuming that they vary proportionally to the respective partition coefficients. This implies the assumption that the intramembrane gas diffusion coefficients are identical for all five gases, which of course may not hold true.

**Table 1:** **Solubility behavior of gases in water and lipids**.

	**CO_2_**	**O_2_**	**NO**	**N_**2**_**	**CO**
Solubility coefficient in H_2_O (25°C); [μmol/l/mmHg]	45^a^	1.7^a^	2.6^b^	0.84^a^	1.3^a^
Solubility coefficient in oil (25°C); [μmol/l/mmHg]	74^c^	6.5^c^		3.8^d^	5.3^e^
Oil-water partition coefficient (25°C)	1.6	3.8		4.5	4.1
Phospholipid-water partition coefficient (25°C)	0.95^f^	3.9^g^	4.4^g^		
Permeability in phospholipid membrane; [cm/s]	~1^h^	4.1^*^	4.6^*^	2.8^**^	2.6^**^

a Bartels et al. (1959);

b Zacharia and Deen (2005);

c Cottonseed oil, CO_2_ temperature-corrected, Lawrence et al. (1946);

d Olive oil, Battino et al. (1984);

e Olive oil at 37°C (temperature effect in oils is minor), Snedden et al. (1996);

f Egg lecithin liposomes, Simon and Gutknecht (1980);

g Egg yolk phosphatidylcholine liposomes, Möller et al. (2005);

h Average from Gutknecht et al. (1977) and Missner et al. (2008).

* Predicted from P_CO_2__ in phospholipid membrane and phospholipid-water partition coefficients.

** *Predicted from P_CO_2__ in phospholipid membrane and oil-water partition coefficients*.

Under the condition of a constant *D*_*M*_, the last line of Table 1 suggests that all gas permeabilities should be rather similar, and all of them should be extremely high. It should be borne in mind, nevertheless, that CO_2_ is unique in Table 1 by possessing absolute solubilities in both water and lipid that are more than an order of magnitude greater than those of the other gases listed.

A note of caution should be added concerning the above value of *P*_CO_2__ in lipid bilayers. In both determinations cited above, that of Gutknecht et al. (1977), who obtained *P*_CO_2__ = 0.35 cm/s, as well as that of Missner et al. (2008), who report *P*_CO_2__ = 3.2 cm/s, it was attempted to eliminate the problem of unstirred layers by special precautions. As points of possible critique it should be mentioned that Gutknecht et al. (1977) showed that apparent bilayer CO_2_ permeability increased with rising concentrations [HCO^−^_3_] + [CO^=^_3_] in the bathing solution (the latter causing increasing facilitation of CO_2_ diffusion in the unstirred layer), and finally reached a plateau value. However, this plateau does not necessarily prove that the diffusion resistance of the unstirred layer has completely vanished as was implied by the authors. Alternatively, the plateau might just indicate that facilitation of CO_2_ diffusion has reached a maximum, which could not be further augmented due to the increasing concentration of the two ions with their inhibitory effects on ionic mobility as well as on carbonic anhydrase activity. Thus, some doubt may remain how free of unstirred layer effects these measurements are. The technique employed by Missner et al. (2008) takes care of the unstirred layer problem by measuring pH gradients (and, derived from them, *p*_CO_2__ gradients) within the unstirred layer in front of the bilayer. However, the extensive theory describing the process observed by these authors required fitting five parameters, one of them *P*_CO_2__, to a single exponential curve of the type shown below (Figure 6A). Moreover, the fitting procedure did not seem to exhibit a clearly defined minimum of the sum of squared deviations, which prompted the authors to introduce a “penalty term” that prevented higher values of *P*_CO_2__ to emerge from the fitting procedure. Thus, both measurements leave some uncertainty about the correct value of *P*_CO_2__ in lipid bilayers. A further problem that is common to both approaches, results from the fact that in both studies planar lipid bilayers were employed, which were made from solutions of the lipids in n-decane. Bunce and Hider (1974) have shown that this may result in membranes whose final composition contains 30% decane. This is expected to lead to elevated *P*_CO_2__ values (a) because decane possesses a two times higher CO_2_ solubility (and partition coefficient) than phospholipid (Simon and Gutknecht, 1980), and (b) because decane may significantly increase membrane fluidity and thus D_M_ for CO_2_ (Gutknecht et al., 1977). On the other hand, Gutknecht et al. (1977) used a 1:1 ratio of phospholipid:cholesterol, which is expected to reduce *P*_CO_2__ (see the following chapter). Missner et al. (2008), in contrast, used planar lipid bilayers with and without cholesterol, and observing no differences, they combined all results to calculate only an overall mean and SD. Nevertheless, in view of the results from other approaches to measure *P*_CO_2__ in phospholipid membranes, as discussed below, a value of ~1 cm/s as a crude estimate of *P*_CO_2__ in lipid bilayers appears reasonable.

In contrast to the high CO_2_ permeabilities obtained by Gutknecht et al. (1977) and Missner et al. (2008), two other groups have obtained extremely low *P*_CO_2__ values from rapid reaction stopped-flow experiments with liposomes. Yang et al. (2000) measured by this technique the CO_2_ permeability of lipid vesicles without and with ~40% cholesterol, obtaining in both cases *P*_CO_2__ ≈ 0.001 cm/s. Prasad et al. (1998) with the same technique also obtained a *P*_CO_2__ of around 0.001 cm/s, no matter whether cholesterol was present or not. Yang et al. (2000) argued that the CO_2_ uptake process of vesicles in the stopped-flow apparatus is probably limited by unstirred layers. We may add that in some commercial stopped-flow apparatuses the mixing is less than perfect, which would further increase the apparent unstirred layer. However, when cells or vesicles rather than just solutions are studied in a stopped-flow experiment, an additional complication arises: when the flow has stopped and cells or vesicles begin to take up some substance, then there is only diffusion, and no convection, that can deliver the substance from the extracellular solution to the membrane. In other words, considerable gradients of this substance can develop in the extracellular/-vesicular space, and the standard assumption of a homogeneous concentration of this substance in the outside solution will not hold. This is not critical, when the uptake process by cells or vesicles is slow, but in a case such as the extremely rapid uptake of CO_2_ into vesicles with a large CO_2_ binding capacity this can become a serious problem.

Indeed, it can be shown that the process of CO_2_ uptake by a vesicle 150 nm in diameter with the buffer capacity of 25 mM HEPES takes <0.1 ms to reach 95% completion, when the chemical reaction inside the vesicles is infinitely fast and when there is no membrane diffusion resistance.

This estimate is based on an approximation by Crank (1957) in combination with the use of the “effective solubility” within the vesicles, a concept applied to red cells by Thews (1960). Accordingly, effective solubility α′ is the amount of CO_2_ plus HCO^−^_3_ taken up by the intravesicular volume per increase in CO_2_ partial pressure. *D* in Crank's treatment is then replaced by *D*′ = D· α/α′, where α is the classical solubility of molecular CO_2_. Crank's expression for 95% completion of the CO_2_ uptake by a sphere then becomes:

$$D^{\prime }\cdot t/r^{2}=0.6,$$

where t is the time required to reach 95% completion, and r is the radius of the sphere. For 25°C, with *D* = 1.8·10^−5^ cm^2^/s, α = 9.8·0^−4^ cm^3^/cm^3^/mmHg, α′ = 53·10^−4^ cm^3^/cm^3^/mmHg (this number being calculated for a *p*_CO_2__ jump from 1 to 40 mmHg), t from the above equation turns out to be 1·10^−5^ s = 0.01 ms.

Thus, without a drastic diffusion resistance of the vesicle membrane to CO_2_, a stopped-flow apparatus with a dead time of a few milliseconds, and perhaps further problems limiting the speed of the process, cannot be expected to capture the kinetics of CO_2_ uptake, provided that substantial intravesicular carbonic anhydrase activity is present and the CO_2_ hydration kinetics assumes a half-time of <1 ms, as was the case in both studies. We conclude therefore that the low *P*_CO_2__ values reported by Prasad et al. (1998) and Yang et al. (2000) are likely to be essentially determined by technical problems of the stopped-flow experiment.

**Figure 1:**
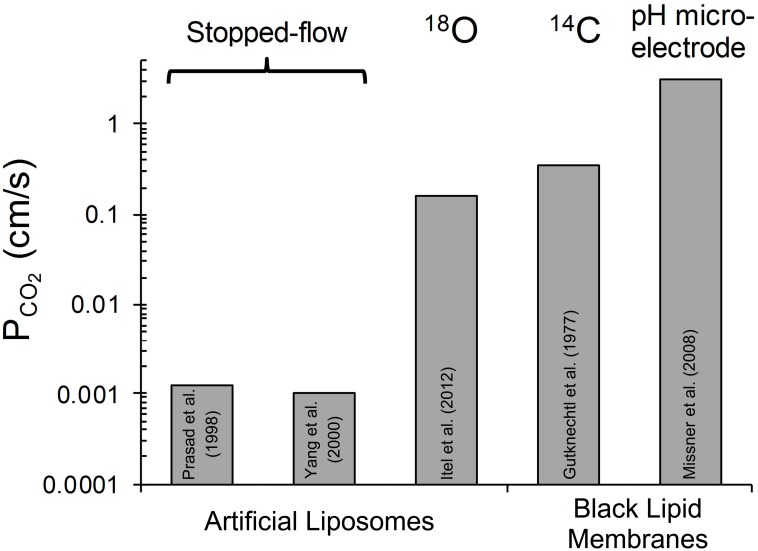
**Overview of CO_2_ permeabilities determined for artificial liposomes or planar lipid bilayers using various techniques**. References see text. ^18^O refers to the ^18^O exchange technique explained in the last section of this article. ^14^C-labeled CO_2_ was used by Gutknecht et al. (1977) to measure steady state transfer of CO_2_. Missner et al. (2008) have used a pH microelectrode to measure the gradient of pH, dpH/dx, in the unstirred layer in front of the membrane to be studied. From this gradient the flux of CO_2_ across the diffusion layer was derived as explained in more detail in section V of this article.

The third approach that has been applied to determine *P*_CO_2__ of artificial liposomes is the mass spectrometric ^18^O exchange technique, which measures very fast processes on an extremely slow time scale (Itel et al., 2012, and see last section of this article). Unstirred layers have been shown to play a minor role (Endeward and Gros, 2009). In the absence of cholesterol, this method gives, as described below, a *P*_CO_2__ > 0.16 cm/s, where this number reflects the upper limit of detectability of the technique for vesicles. Summarizing the discussion of this paragraph, it appears to be the best guess at present that the true *P*_CO_2__ of cholesterol-free lipid bilayers is certainly >0.16 and probably around 1 cm/s. Figure 1 gives a summary of the *P*_CO_2__ values obtained for artificial lipid bilayers, by the stopped-flow technique using vesicles, by observing steady state CO_2_ transfer across planar lipid bilayers (Gutknecht et al., 1977; Missner et al., 2008), or by ^18^O mass spectrometry applied to vesicles (Itel et al., 2012). The drastic difference between results from stopped-flow experiments and those from the other techniques is obvious.

Recent work has called into question the classical simple view that cellular gas permeability is extremely high and reflects essentially the lipid/water partition behavior of the gas, a view that has recently, for example, been re-stated by Missner and Pohl (2009). In the case of CO_2_ several alternative mechanisms are currently being discussed. In the following sections, we will critically discuss the concepts presently proposed.

## Effect of cholesterol on CO_2_ permeability of membranes

**Figure 2:**
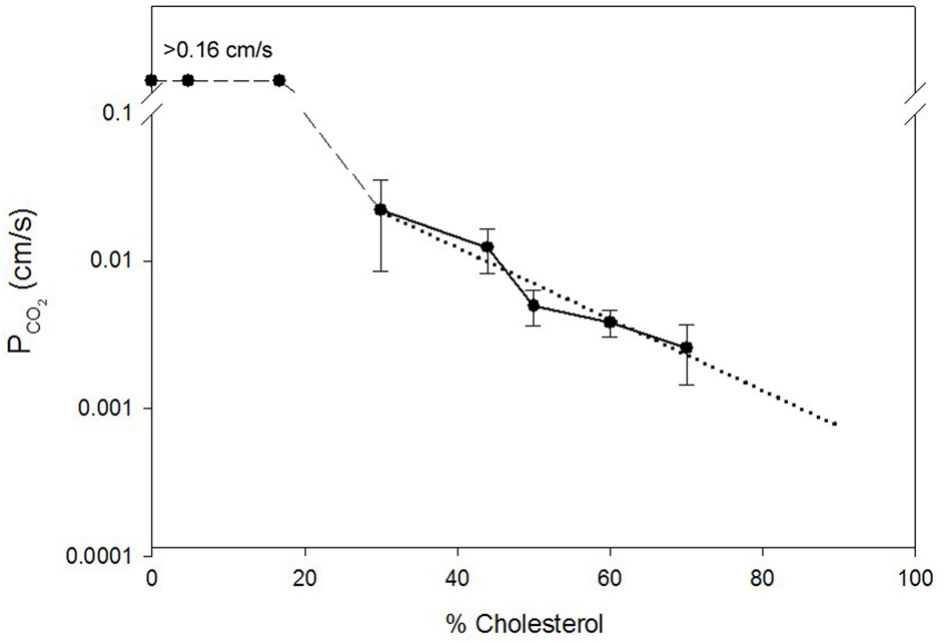
**Dependence of the CO_2_ permeability of phospholipid vesicles on their cholesterol content**. The latter is given on the x-axis in mol% per total membrane lipid. Vesicle phospholipid composition was phospatidylcholine:phosphatidylserine = 8:2. *P*_CO_2__ was determined by the mass spectrometric ^18^O exchange technique. Dotted line is the linear regression line for all data points between 30 and 70% cholesterol. *P*_CO_2__ values between 0 and 17% cholesterol were above the upper limit of detectability of the method of 0.16 cm/s. Figure reproduced from Itel et al. (2012), with permission.

Missner et al. (2008) as well as Prasad et al. (1998) and Yang et al. (2000) have observed no effect of cholesterol on *P*_CO_2__ of artificial lipid membranes. Possible limitations of Missner's experimental approach, e.g., the presence of decane in their planar bilayers and problems with their fitting procedure, have been discussed above. Likewise, it appears probable that an indequate time resolution of the stopped-flow experiments employed by Prasad et al. (1998) and by Yang et al. (2000) slowed down the observed kinetics so drastically that any moderate changes in true membrane permeability could not become apparent. In view of this it is interesting that Itel et al. (2012) have recently reported that cholesterol has a most drastic effect on the CO_2_ permeability of phospholipid vesicles. This is illustrated in Figure 2, which is reproduced from their paper. It can be seen that between 0 and 30 mol% cholesterol content of the vesicles *P*_CO_2__ falls from >0.16 cm/s to ~0.02 cm/s, i.e., by at least one order of magnitude. If *P*_CO_2__ at 0% cholesterol were 1 cm/s as given in Table 1, this fall would even occur over two orders of magnitude. Between 30 and 70 mol% cholesterol then, *P*_CO_2__ decreases by another order of magnitude to ~0.0024 cm/s. It was concluded that cholesterol between 0 and 70% can reduce vesicle *P*_CO_2__ by 2–3 orders of magnitude. This drastic effect of cholesterol does not seem to be due to a decrease in CO_2_ solubility in the lipid phase, as Simon and Gutknecht (1980) report only a 25% reduction of CO_2_ solubility when 50% cholesterol is added to egg lecithin in liposomes. Thus, cholesterol is likely to cause a significant reduction of intramembrane CO_2_ diffusivity *D*_*M*_ [see Equation (4)].

It is very important to note that the results of Figure 2 are in excellent agreement with the molecular dynamics simulations of Hub et al. (2010), who also predict that cholesterol can reduce the CO_2_ permeability of lipid bilayers by several orders of magnitude. Itel et al. (2012) have confirmed the quantitative findings of Figure 2 by a qualitative approach applied to intact cells. Decreasing the cholesterol content of MDCK cells by cholesterol depletion with β-cyclodextrin caused a dramatic ≥40-fold increase in CO_2_ permeability. Inversely, enrichment of these membranes by cholesterol-preloaded β-cyclodextrin reduced *P*_CO_2__ to 1/3 of normal MDCK cells.

Does the finding shown in Figure 2 for artificial vesicles also apply to cell membranes? Itel et al. (2012) have shown that the CO_2_ permeability of cell membranes that are devoid of protein gas channels, as will be discussed below in chapter VI of this article, can be fairly precisely predicted from their cholesterol content, when the dotted regression line of Figure 2 is used. Thus, it was concluded that also in cell membranes, in the absence of gas channels, cholesterol is the decisive determinant of CO_2_ permeability and therefore is much more important than the exact phospholipid composition and membrane protein content. Since the membranes of many cells possess a cholesterol content of around 40%, a *P*_CO_2__ of close to 0.01 cm/s is predicted from the regression line of Figure 2. Indeed, Itel et al. (2012) report for all of four cell types, namely two gas channel-free cell lines (MDCK and tsA201 cells), the red cell membrane without functional gas channels, and the presumably gas channel-free basolateral membrane of proximal colon epithelial cells, *P*_CO_2__ values of around 0.01 cm/s. In conclusion, the permeability of many cell membranes is two orders of magnitude lower than the *P*_CO_2__ value of cholesterol-free artificial lipid membranes, which has long been believed to be representative for cell membranes too.

## “CO_2_-impermeable” membranes

A very early report of a cell membrane poorly permeable to a gas describes the observation of Kikeri et al. (1989) that the apical membranes in the medullary thick ascending limb of Henle in mouse kidney are “impermeable” to NH_3_. A few years later several instances of membranes impermeable to CO_2_ were reported, always apical membranes of epithelial cells. Waisbren et al. (1994) observed an apparent impermeability for CO_2_ of the apical epithelial chief and parietal cell membranes in isolated perfused gastric glands. Hasselblatt et al. (2000) reported the same property for the apical epithelial membranes of *in vitro* perfused rat colonic crypts. Endeward and Gros (2005) then made an analogous observation on the surface cells of guinea pig colon epithelium, and for the first time they were able to quantitate this “impermeability”; they determined by ^18^O exchange mass spectrometry the CO_2_ permeability of the apical proximal as well as distal colonic epithelial membrane to be ~0.001 cm/s. This was 10 times lower than the CO_2_ permeability of several gas channel-free cell membranes as discussed above. The mechanism of the very low CO_2_ (and NH_3_) permeability of several apical epithelial membranes was entirely unclear, and only recently the results of Itel et al. (2012) have elucidated the cause of this property. Quite fortunately, Meyer zu Düttingdorf et al. (1999) have been successful in obtaining a good separation of apical and basolateral membranes of guinea pig colon epithelium and they performed an analysis of their lipid composition. They found a cholesterol content of 42% in the basolateral membrane of the proximal colon, which is what many cells have, but as much as 77% cholesterol in the apical membrane. Using the regression line of Figure 2 one predicts for this cholesterol content a CO_2_ permeability close to 0.001 cm/s. This means that the very low permeability of this membrane can be satisfactorily explained simply by its cholesterol content. We conclude that cells can regulate their CO_2_ permeability over a wide range (and likely their NH_3_ permeability as well) by altering the cholesterol content of their membrane.

The physiological advantage of making cell membranes impermeable to gas is obvious in the case of NH_3_, where the potentially very high partial pressures in the lumen of the stomach as well as the colon would be toxic to the epithelial cells if the NH_3_ had access to their interior. Likewise, the potentially very high CO_2_ partial pressures (up to 0.5 atm) in both lumina would constitute a severe acid load for the cells, if the CO_2_ were able to enter them (Endeward and Gros, 2005).

## Which CO_2_ permeability is necessary for respiring cells?

**Figure 3:**
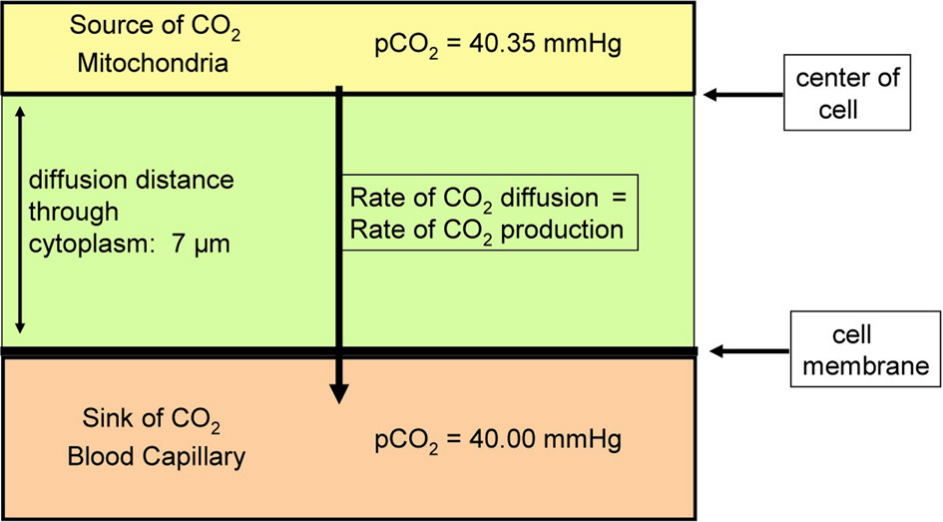
**Scheme of the model used to estimate the effect of membrane CO_2_ permeability on the release of CO_2_ from a cardiomyocyte**. CO_2_ diffuses from the CO_2_ source located in the center of the cell first through the cytoplasm, and then through the plasma membrane, into the blood capillary.

Itel et al. (2012) have reported *P*_CO_2__ values of 0.01 cm/s for two cell lines. Cells in culture usually exhibit a low oxidative metabolism, as they are not in an active state and often cover part of their energy requirement by anaerobic metabolism. Would a permeability of 0.01 cm/s suffice, for example, for highly active cells such as cardiomyocytes *in vivo* under conditions of heavy exercise? We present in the following calculations an answer to this question, employing a simplified model of CO_2_ release from a cardiomyocyte. The cardiomyocyte is considered as a cuboid, which in conjunction with further simplification allows us to model CO_2_ release on the basis of diffusion across a plane sheet. As the radius of human cardiomyocytes is 7 μm, we approximate the geometry of the cell by a plane sheet of 14 μm thickness, and perform the calculation for the half-thickness of the cuboid of 7 μm (Armstrong et al., 1998; Endeward et al., 2010; Endeward, 2012). In Figure 3, the upper border of the cytoplasmic space then represents the center of the cell, where for simplicity the source of CO_2_, i.e., the mitochondria, is assumed to be located. From there the CO_2_ diffuses through the cytoplasm over 7 μm, and then diffuses through the cell membrane before it is taken up by the blood in the capillary. We consider steady state conditions, so the CO_2_ partial pressures in the center of the cell and in the capillary are constant.

**Figure 4:**
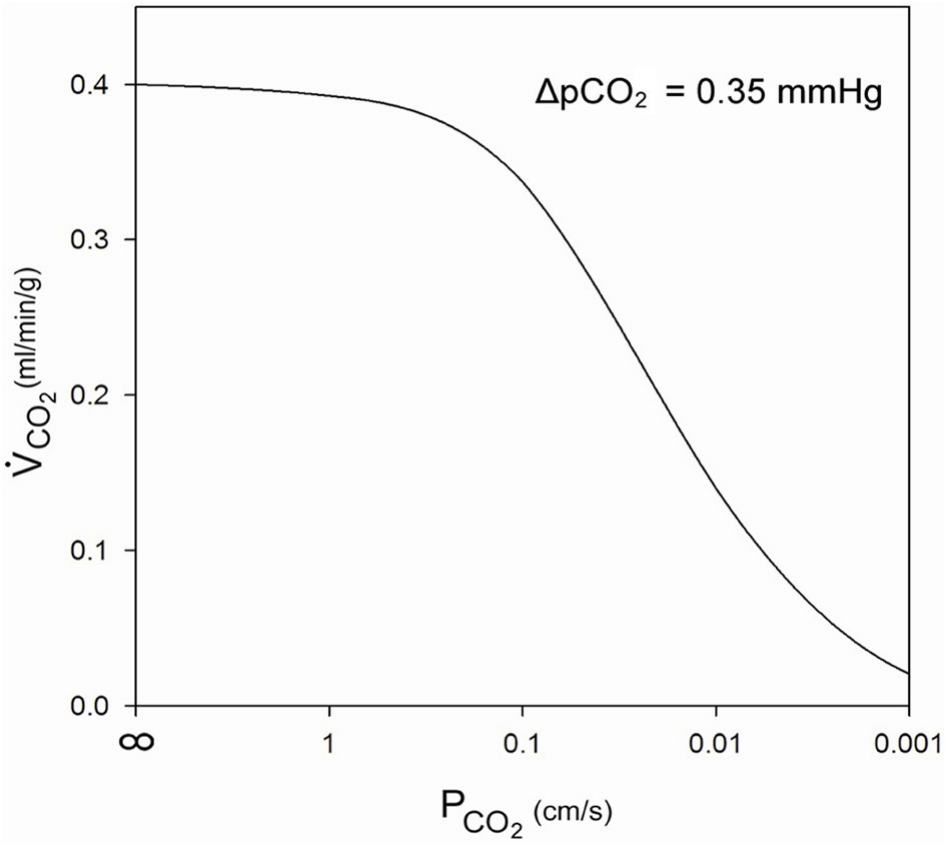
**Influence of membrane CO_2_ permeability on CO_2_ release from a maximally metabolizing cardiomyocyte at a given partial pressure gradient of 0.35 mmHg over the half-thickness of the cell**.

What is a realistic *p*_CO_2__ gradient within the cell? It is generally accepted that *p*_CO_2__ gradients in tissues—in contrast to *p*_O_2__ gradients—are extremely small, but no information on their precise value is available. Therefore, we use here the following approach. For the condition of heavy exercise, the heart can assume an O_2_ consumption, and about identical CO_2_ production, of 0.4 ml gas/g tissue/min (Endeward et al., 2010). With the known cytoplasmic CO_2_ diffusion coefficient, 1.3 · 10^−5^ cm^2^/s, and the diffusion path given in Figure 3, we calculate the partial pressure difference, Δ*p*_CO_2__, necessary to produce a flow of CO_2_ of 0.4 ml CO_2_/g tissue/min from CO_2_ source to CO_2_ sink. In this calculation, the cell membrane is assumed to offer no diffusion resistance to CO_2_ at all. This yields a necessary Δ*p*_CO_2__ of 0.35 mmHg as indicated in Figure 3. This is a reasonable number in view of the expected small CO_2_ gradients in tissues. We then add the diffusion resistance of the membrane to that of the 7 μm thick cytoplasmic layer, where the latter amounts to 54 s/cm. Using Fick's first law of diffusion, the rate of CO_2_ production, ${\dot{\text{V}}}_{{\text{CO}}_{\text{2}}}$, and of diffusion from the CO_2_ source to the capillary, is then given by:

(5) $${\dot{\text{V}}}_{{\text{CO}}_{\text{2}}}={\left({\text{d/D}}_{{\text{CO}}_{\text{2}}}+1/P_{{\text{CO}}_{\text{2}}}\right)}^{-1}\cdot \text{A}\cdot \alpha_{{\text{CO}}_{\text{2}}}\cdot \Delta p_{{\text{CO}}_{2}}/(\text{d}\cdot \text{A}),$$

where the terms in the first brackets represent the sum of the resistances of diffusion through the cytoplasm and across the membrane. d is the cytoplasmic diffusion path, D_CO_2__ the cytoplasmic CO_2_ diffusion coefficient, *P*_CO_2__ membrane CO_2_ permeability, A the unit diffusion area (1 cm^2^), α_CO_2__ the solubility of CO_2_ in tissue (7.2·10^−4^ mm Hg^−1^), and the product d·A is the volume of cells in which ${\dot{\text{V}}}_{{\text{CO}}_{\text{2}}}$ is produced. This equation leads to the curve shown in Figure 4. At *P*_CO_2__ = ∞ the value of ${\dot{\text{V}}}_{{\text{CO}}_{\text{2}}}$ is, as explained above, the maximal rate of cardiac CO_2_ elimination of 0.4 ml/min/g. At constant Δ*p*_CO_2__, the CO_2_ production rate decreases with decreasing *P*_CO_2__. While a *P*_CO_2__ of 1 cm/s has only a small limiting effect on the rate of CO_2_ release, the *P*_CO_2__ found in cells in culture, 0.01 cm/s, would reduce CO_2_ production/release to 1/3 of the value in the absence of a membrane resistance. *P*_CO_2__ = 0.001 cm/s would even reduce it to 1/20. Figure 4 shows that *P*_CO_2__ values ≤0.1 cm/s become increasingly critical for cellular CO_2_ release, an effect that can only be overcome by raising intracellular *p*_CO_2__, and thus Δ*p*_CO_2__ [see Equation (5)]. It can also be deduced from Equation (5) that a lower rate of CO_2_ production, for example that of 0.003 ml/g/min as it occurs in resting skeletal muscle, which is only 1% of the value in maximally working heart, can at identical or lower Δ*p*_CO_2__ easily be achieved without any limitation with a *P*_CO_2__ of 0.01 cm/s, even with *P*_CO_2__ = 0.001 cm/s a Δ*p*_CO_2__ of 1/5 of 0.35 mmHg would suffice. Thus, it is clear that, while *P*_CO_2__ = 0.01 cm/s is more than adequate for cells with low oxidative metabolism, a >10 times higher *P*_CO_2__ would seem important for cells with an extremely high oxygen consumption. It remains to be tested, whether such cells indeed exhibit CO_2_ permeabilities of that size.

A different approach to the same question has been reported by Endeward et al. (2008), who evaluated the effect of membrane CO_2_ permeability on the kinetics of gas exchange of red cells. Human red cells normally possess a CO_2_ permeability of 0.15 cm/s. With this value, the time required for 95% completion of the release of CO_2_ as it occurs in the lung was calculated to be 110 ms, which may be compared to the capillary transit time in the lung of 700 ms. If red cells without functional gas channels, which have a *P*_CO_2__ of 0.01 cm/s (Endeward et al., 2008), are considered, the required time rises to 1000 ms, thus exceeding the available transit time in the lung. The limitation of CO_2_ release is expected to be more pronounced under conditions of exercise, when the transit time can fall to 350 ms. The problem caused by such a low *P*_CO_2__ can only be overcome by increasing the *p*_CO_2__ gradient across the alveolar diffusion barrier, and it is clear that the normal high CO_2_ permeability of red cells will ensure a maximally economical CO_2_ release in the lung.

We conclude from these two approaches that optimal CO_2_ release in cells with very high rates of gas exchange, like cardiomyocytes and red cells, requires high CO_2_ permeabilities >0.1 cm/s.

## Is intracellular carbonic anhydrase responsible for the high CO_2_ permeability of some cells?

Recently, Zocher et al. (2012) proposed that the low permeability of the urinary bladder with a *P*_CO_2__ of ~0.00016 cm/s is not due to the uroplakin of the bladder epithelium but to the absence of carbonic anhydrase in that epithelium, a hypothesis constituting a basically new concept of membrane CO_2_ transfer. Measurements of CO_2_ transfer across monolayers of MDCK cells, which are known to possess intracellular carbonic anhydrase, seemed to confirm this hypothesis: (1) when Zocher et al. (2012) added the carbonic anhydrase inhibitor acetazolamide to the solutions to inhibit all carbonic anhydrase (intra- as well as extracellular), the CO_2_ flux across the epithelium was very low, (2) it increased moderately when no acetazolamide was added, and (3) it increased 14-fold in comparison to experiment (1) when they added a very high activity of carbonic anhydrase (as produced by 2 mg CA/ml) to the solutions of the bathing medium of the epithelium. They concluded from this that “carbonic anhydrase expression may uniquely regulate the tightness of biological membranes to CO_2_” and that their results “rule out that aquaporins significantly contribute to the maintenance of acid base homoeostasis.” Thus, these authors propose a biological mechanism that would raise the CO_2_ permeability of a membrane not by reducing membrane cholesterol, as discussed above, nor by insertion protein gas channels into the membrane, as we will discuss below.

**Figure 5:**
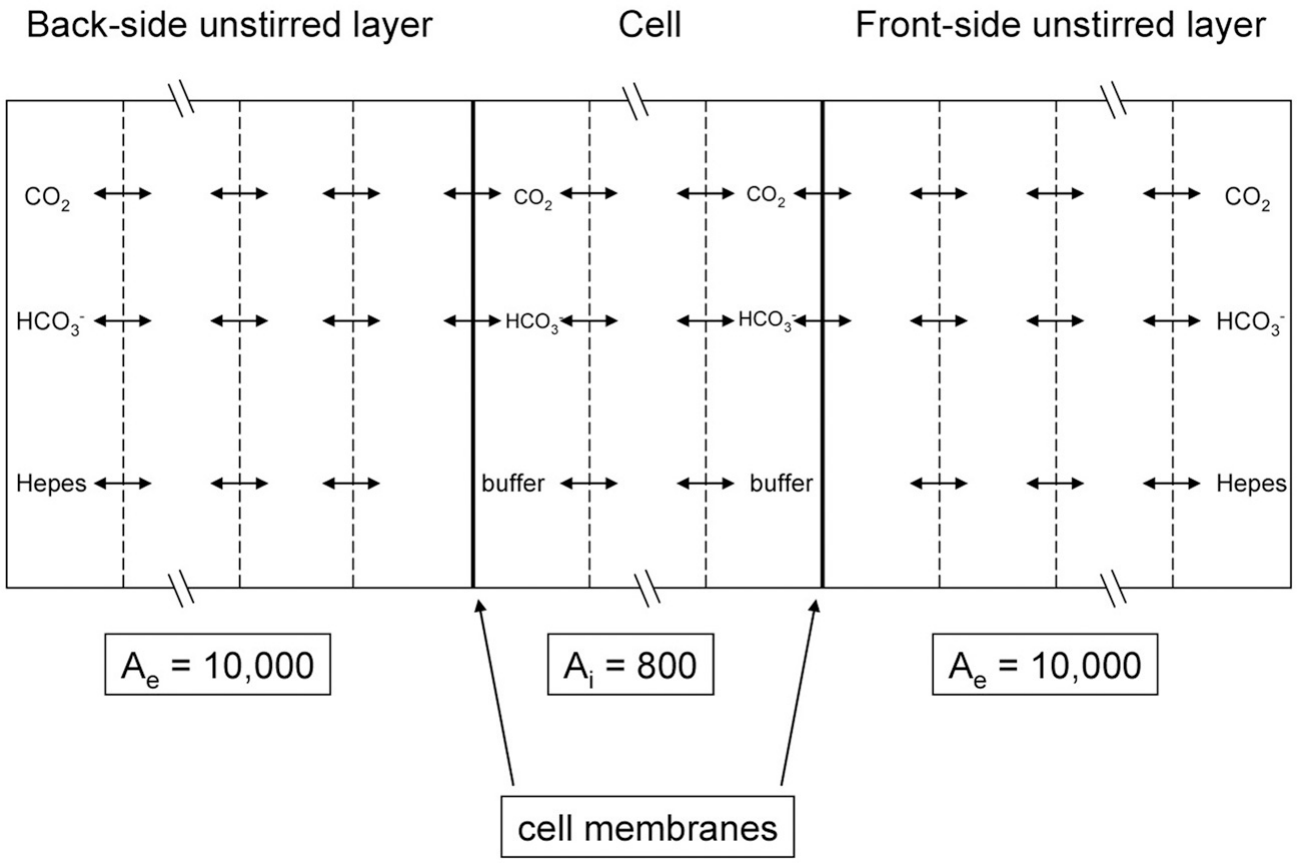
**Scheme of the mathematical model used to simulate the CO_2_ flux measurements of Zocher et al. (2012)**. MDCK cell monolayers were exposed to an extracellular solution on the apical (back) side as well as on the basal (front) side. The flow is determined by measuring the pH gradient with a pH microelectrode moving across the front-side unstirred layer and by inserting this pH gradient into Equation (6).

**Figure 6:**
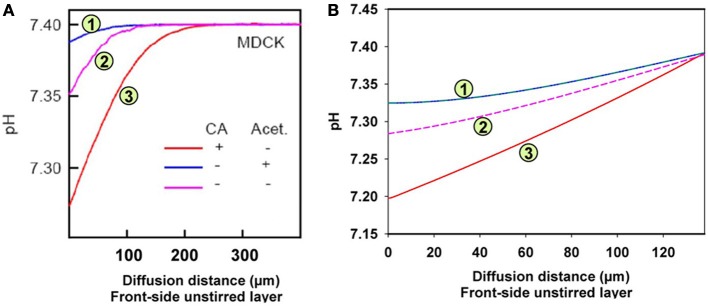
**pH gradients in the unstirred layer in front of the epithelial layer of MDCK cells**. Acidification relative to the pH in the bulk extracellular solution occurs due to the CO_2_ influx from the epithelium. **(A)** Measured pH profiles reproduced with modification from Figure 7A of Zocher et al. (2012), with CC-BY license. Acet. = acetazolamide, **(B)** calculated pH profiles using the model given in Figure 5.

In order to understand the mechanism underlying the results of Zocher et al. (2012), we modeled the experimental situation used in their experiments with MDCK monolayers. The principle of their measurements was to expose a layer of epithelium to a *p*_CO_2__ gradient, and then measure the pH gradient that exists after steady-state has been reached in the unstirred layer between the front side of the epithelium (see Figure 5) and the bulk solution in the downstream compartment. They do this by moving a pH microelectrode from the bulk solution towards the surface of the epithelium and recording pH as a function of the distance from the epithelial surface. Figure 6A shows examples in a figure reproduced from their paper (Zocher et al., 2012). Up to this step their procedure is identical to that used by Missner et al. (2008), which has been discussed above. However, they avoid the extensive theory required by Missner's approach by using an elegant approximation in the evaluation of these experiments. Zocher et al. (2012) use the first linear parts of the curves of Figure 6A to calculate the flux of CO_2_ across the epithelium, roughly approximated by the HCO^−^_3_ flux = (buffer-facilitated) H^+^ flux through the unstirred layer in front of the epithelium:

(6) $$\begin{array}{l} \text{transepithelial }{\text{CO}}_{2}\text{ flux}\approx \text{buffer-facilitated proton flux in}\\ \text{ unstirred layer}\\ \;={\text{D}}_{\text{B}}\cdot \text{BF}\cdot \Delta \text{pH}/\Delta \text{x}, \end{array}$$

where D_*B*_ is the diffusion coefficient of the buffer present in the extracellular solution, BF is the buffer factor provided by this buffer (in mM/ΔpH), and ΔpH/Δx is the linear initial slope of the curves in Figure 6A.

Figure 5 illustrates the experimental situation employed by Zocher et al. (2012) as well as the principle of the present mathematical model. Our model considers two unstirred layers on either side of the epithelial layer, plus the intracellular compartment. At the left hand side of the back-side unstirred layer and at the right-hand side of the front-side unstirred layer, the conditions of the bulk extracellular solution are fixed: pH = 7.4 in NaCl-HEPES buffer with 25 mM bicarbonate. *p*_CO_2__ at the back side was then increased abruptly in the model by raising [HCO^−^_3_] from 25 to 50 mM in the left-hand side bulk solution, and subsequently pH at the front-side became acidified by the ensuing flux of CO_2_ across the epithelium. In all three compartments we modeled the transport of CO_2_, HCO^−^_3_, H^+^, and HEPES (as a H^+^-carrier) by considering all diffusion and reaction processes involved. The membranes were considered impermeable to HCO^−^_3_ and were assigned a CO_2_ permeability of 0.01 cm/s (Itel et al., 2012). The differential equations were solved by a finite difference method as implemented in MATLAB 2008b. Each of the three compartments of Figure 5 was divided into 50 volume elements for this calculation.

Figure 6A shows the experimental results obtained by Zocher et al. (2012). As described above, these authors used the initial slopes of the curves in Figure 6A to calculate the approximate CO_2_ fluxes occurring across the monolayer of MDCK cells. From these slopes as read from the curves of Figure 6A, the flux resulting in curve (3; red) is estimated to be 6.3 times greater than the flux leading to curve (1; blue). This was interpreted to mean that in the presence of intra- (as well as extracellular) CA the epithelium possesses a higher CO_2_ permeability, and when no extracellular CA is added and all the endogenous CA of the MDCK cells is inhibited by acetazolamide, the CO_2_ permeability is 6.3 times lower. In the absence of the CA inhibitor, the curve (2; purple) assumes an intermediate position due to the endogenous CA activity of the cells. The conclusion was that carbonic anhydrase, intracellular and ostensibly also extracellular, substantially increases CO_2_ permeability of the cell layer, which was attributed to an increase in cell membrane *P*_CO_2__ by Zocher et al. (2012).

Figure 6B shows the results of the present model calculations. In contrast to Figure 6A, we consider the unstirred layer only, without the bulk solution, and assume the unstirred layer to extend over 130 μm in front of the MDCK cell layer. At the outer end of this unstirred layer (right hand side in Figure 6B), we hold the conditions of the extracellular bulk solution fixed (pH = 7.4, [HCO^−^_3_] = 25 mM). Reassuringly, our model predicts a similar about eight times greater initial slope of curve (3; red) than of curve (1; blue), where in analogy to Figure 6A, curve (3) was calculated for an extracellular CA activity A_CA_,e of 10,000 and an intracellular activity A_CA_,i of 800, which is realistic for MDCK cells (Al-Samir et al., 2013). In contrast, curve (1) was calculated for the complete absence of CA in the extra- as well as the intracellular space, which corresponds with the addition of 1 mM acetazolamide in Zocher's experiments. We conclude, that the model of Figure 5 nicely explains curves (1) and (3) of their experiment as it shows a similar difference in pH slopes to that observed by them. What is the mechanism of the intermediate (~4-fold) increase in slope of their curve (2) over that of curve (1)? Curve (2) of Figure 6A was obtained without any additions at all. A four-fold acceleration of the intracellular CO_2_ flux by facilitated CO_2_ diffusion due to A_CA_,i is unlikely in view of the findings that facilitated diffusion inside red cells as well as inside skeletal muscle (Gros and Moll, 1972; Kawashiro and Scheid, 1976) accelerates intracellular CO_2_ transport by no more than 60–80%. We have speculated therefore, that the slope of curve (2) in Figure 6A may be increased due to the combined action of intracellular and a small amount of membrane-bound extracellular CA, which both occur in MDCK cells (Pfaller et al., 1989). Modeling this with an intracellular CA activity A_CA_,i of 800 and an assumed membrane-bound surface-associated extracellular activity A_CA_,s of 100, we obtain curve (2) in Figure 6B, whose slope is indeed intermediate between those of curves (1) and (3). We note that in this model the activity A_CA_,s = 100 is only applied in the one volume element immediately adjacent to the cell membrane, while CA activity is assumed to be absent from all other volume elements of the unstirred layers. In conclusion, our model satisfactorily explains all experimental results of Zocher et al. (2012) as shown in Figure 6A for MDCK cells.

Nevertheless, the present model does not confirm the conclusion of Zocher et al. (2012) that the flux of CO_2_ across the cell layer depends strongly on CA activity. This is due to the basal assumption of their method, which is that the CO_2_ flux leaving the cell layer and entering the right-hand side extracellular unstirred layer compartment (Figure 5), can in all cases be deduced from the surface pH gradient and the proton flux derived from it by Equation (6). Such a calculation is only a reasonable approximation, however, if the extracellular carbonic anhydrase activity is very high and therefore the CO_2_-HCO^−^_3_-H^+^-system is in near-complete chemical equilibrium. A CAII (the isoform II of CA) concentration of 2 mg/ml in the NaCl-HEPES solution, which was used experimentally, is more than the CAII concentration present in human red cells, and therefore the activity of 10,000 as used in our model is a minimum estimate of the CA activity in that solution (Wistrand, 1981; Endeward et al., 2006a,b; Al-Samir et al., 2013). Thus, the assumption of near-perfect chemical equilibrium is certainly justified for curves (3) in Figures 6A,B. The CO_2_ fluxes derived from these slopes should be correct (and in Figure 6B indeed come close to the true ones, as derived from the model and given in Table 2, for the case of curve 3). However, in the absence of any CA activity, as in curve (1) of Figure 6A, and also in the presence of only very limited extracellular CA activity as is likely the case in curve (2) of Figure 6A, it is not permissible to derive CO_2_ fluxes from the pH gradient. When the speed of the CO_2_ hydration reaction is slow, the CO_2_ leaving the cell layer will not react fast enough to produce the amount of protons that would be produced in the presence of high CA activity, and the CO_2_-HCO^−^_3_-H^+^-system will remain far from chemical equilibrium. Thus, deriving CO_2_ fluxes from the extracellular pH profile using Equation (6) in the manner described, will lead to a substantial underestimation of these fluxes in the cases of curve (2), and even more so of curve (1). This is illustrated in Table 2, which gives the CO_2_ fluxes calculated from the mathematical model of Figure 5. It is apparent that that these fluxes do not vary by a factor of 8, as the initial pH slopes do, or even 14, which is the average factor of increase in CO_2_ flux given by Zocher et al. (2012). Instead, while the fluxes do show some minor positive correlation with extracellular CA activity, this effect does not exceed an increase in CO_2_ flux by more than 25%. This small effect is due to the acceleration of CO_2_ hydration on the membrane surface, which will remove the CO_2_ just having crossed the membrane more rapidly from the extracellular surface region of the cell membrane, thus maintaining a somewhat larger gradient of CO_2_ across the membrane. This of course does not reflect any alteration of the CO_2_ permeability properties of cellular membranes. We conclude that carbonic anhydrase is not involved in regulating the CO_2_ permeability of cell membranes *per se*, and other mechanisms must be responsible for the considerable variations of CO_2_ permeability found between different membranes (Itel et al., 2012).

**Table 2:** **Summary of the conditions in the experiments with MDCK monolayers by Zocher et al. (2012) and in the model of Figure 5**.

	**Experimental condition**	**CA activities used in the model, and likely present also in experiment**			**Initial pH slope in front-side unstirred layer (from model, Figure 6B) (pH/cm)**	**True CO_2_ flux derived from model (Figure 6B) (mmoles/cm^2^/s)**
		A_CA_,i	A_CA_,e	A_CA_,s		
Curve 1	1 mM acetazolamide	–	–	–	1.6	2.0 · 10^−6^
Curve 2	No additions	800	–	100	5.1	2.2 · 10^−6^
Curve 3	2 mg/ml CA in extracelluar buffer	800	10,000	–	12.6	2.5 · 10^−6^

A_CA_,i intracellular CA activity, A_CA_,e extracellular activity in the bathing solutions, A_CA_,s assumed value of membrane-bound, cell surface-associated CA activity, which is active only in a small volume element immediately adjacent to the cell membrane. The CO_2_ fluxes in the rightmost column are derived from the mathematical model, and for curves 1 and 2 deviate substantially from those obtained from the slopes of the curves in Figure 6B (2nd column from right). If it were permissible to consider the slopes of the 2nd column from right as proportional to the CO_2_ fluxes, an 8-fold decrease in flux under the condition of curve 1 would be predicted in comparison to the condition of curve 3.

## Protein channels for molecular CO_2_ in membranes of high CO_2_ permeability

While “gas-impermeable” membranes have been known for almost 25 years, it is only 15 years ago that the first reports suggesting the existence of protein gas channels have appeared. In the first part of this chapter we will discuss **studies that have been in favor of the function of AQP1 and other proteins as channels for CO_2_**:

1. Nakhoul et al. (1998) reported that expression of aquaporin1 (AQP1) in *Xenopus* oocytes increases the rate of intracellular acidification seen upon exposure of the oocyte to an increase in extracellular *p*_CO__2_. They proposed that AQP1 acts as a CO_2_ channel.
2. Forster et al. (1998) in the same year reported that the effect of DIDS (4,4′- diisothiocyano-2,2′-stilbenedisulfonic acid) on the C^18^O^16^O-exchange kinetics in human red cells as observed by mass spectrometry cannot be explained solely by inhibition of the anion exchanger 1. When applying the mathematical model that describes this process in terms not only of CA activity and HCO^−^_3_ permeability, but also of CO_2_ permeability, as first introduced by Wunder and Gros (1997, 1998) and Wunder et al. (1998), they concluded that DIDS is a powerful inhibitor of red cell membrane CO_2_ permeability. Since DIDS is an ε-amino group reagent affecting a variety of proteins, they concluded that CO_2_ permeation in the red cell membrane must be a process mediated by a membrane protein.
3. Again in the same year, Prasad et al. (1998) reported that reconstitution of AQP1 into liposomes increased their CO_2_ permeability as determined from stopped-flow experiments. However, as discussed above, the absolute values of the CO_2_ permeabilities these authors obtained (10^−3^ cm/s) suggests that technical problems with the stopped-flow technique caused a problem in these measurements. Actually, the same group later on argued very clearly against a role of AQP1 as a channel for CO_2_ (Missner et al., 2008; Zocher et al., 2012). So these results should actually not be used as an argument in favor of gas channels.
4. An important argument in favor of aquaporin's role as a CO_2_ channel came more recently from the work of Uehlein et al. (2003). They showed that the tobacco aquaporin NtAQP1, like AQP1 (Nakhoul et al., 1998), increases CO_2_ permeation when expressed in *Xenopus* oocytes. Moreover, NtAQP1 was shown in plants to increase both cellular water and CO_2_ permeability and promote leaf growth. Thus, the authors showed in plants for the first time a clear physiological function of a protein CO_2_ channel.

In the years 2006–2008 Endeward et al. (2006a,b, 2008) elucidated the mechanism of CO_2_ permeation across the human red cell membrane. In these studies, using blood from AQP1- and Rh-deficient persons, they were the first to detect a second CO_2_ channel in addition to AQP1, namely the Rhesus protein RhAG (Rhesus-associated glycoprotein), which was already known to conduct ammonia (Ripoche et al., 2004). AQP1 and RhAG each contribute one half of the normal CO_2_ permeability of this membrane, which amounts to *P*_CO_2__ = 0.15 cm/s, about 10 times greater than the permeability in the functional absence of the two channels. Both channels are efficiently though not quite completely inhibited by DIDS, explaining the strong effect of this inhibitor on red cell *P*_CO_2__ and thus the earlier finding by Forster et al. (1998). The CO_2_ conductance through AQP1 was in addition strongly inhibitable by the mercury reagent pCMBS (p-chloromercuribenzene sulfonate) that is known to effectively inhibit the aquaporin water channel (Echevarria et al., 1993). In the Rh protein mediating CO_2_ transport, Endeward et al. (2008) demonstrated a competition between NH_3_ and CO_2_, which was a strong argument for RhAG being the CO_2_ channel, rather than other members of the Rhesus protein complex in the red cell membrane.

The evidence in favor of CO_2_ channels listed so far has mostly been obtained on specialized membranes such as those of red cells and oocytes. Therefore, it was of interest to extend this to other membranes. Itel et al. (2012) have recently expanded the range of membranes exhibiting CO_2_ conductance by aquaporin 1 by (a) incorporating AQP1 into artificial lipid bilayers containing 50% cholesterol and (b) expressing AQP1 in MDCK cells that contain about 40% cholesterol (see discussion below).

The role of AQP1 as a CO_2_ channel was supported by molecular dynamics simulations by Hub and de Groot (2006) and by Wang et al. (2007), who both predicted that AQP1 lets pass molecular CO_2_, through the water channel of the AQP1 monomer, and perhaps mainly through the central pore that exists in the AQP1 tetramer between the four monomers. The authors' reservation against a possible physiological significance of the CO_2_ channel came from their observation that the lipid bilayer surrounding the AQP tetramers was even more permeable to CO_2_ than AQP. Very recently, Uehlein et al. (2012) have provided an especially convincing piece of experimental evidence for AQP being indeed a channel for CO_2_. They incorporated the tobacco NtAQP1 into a planar layer of gas-tight triblock-copolymers. While the untreated membrane shows no CO_2_ permeation whatsoever, incorporation of the NtAQP1 induces a most impressive permeability for CO_2_. Hub et al. (2010) confirmed by molecular dynamics calculations that the Rhesus protein Rh50, a member of the Amt/MEP/Rh family, indeed can serve as a pathway for CO_2_ in addition to NH_3_, thus confirming the experimental results on RhAG by Endeward et al. (2006b, 2008).

Musa-Aziz et al. (2009) and Geyer et al. (2013a) showed, by expression in oocytes, that several members of the aquaporin family 0–9 besides AQP1 are good conductors of CO_2_. The authors take the amplitudes of the (alkaline) surface pH transients ΔpH_S_ that appear upon exposure of the oocytes to CO_2_ as a semiquantitative measure of the aquaporin-induced CO_2_ permeability. Somersalo et al. (2012) presented an extensive theory deriving the *P*_CO_2__ of the oocyte from the value of the transient ΔpH_S_ peak. Somersalo et al. (2012) point out the limitations of this measure of *P*_CO_2__ to be due to (a) the the peak value of the pH surface transient not being linearly related to *P*_CO_2__, (b) the blunt surface pH electrode being pushed up tightly against the vitelline membrane of the oocyte, which creates a limited unstirred space there (of a size not precisely known) and causes the pH transient sensed by the electrode to be likely different from what it is in the remainder of the oocyte surface, and (c) the presence of the vitelline membrane *per se* being expected to create an additional unstirred space on the oocyte cell membrane, whose influence is hard to assess. Thus, comparisons of these pH transients caused by different aquaporins expressed in oocytes will be possible and provide most valuable insights, but will be semiquantative rather than quantitative. With this approach, the mentioned group finds ΔpH_S_ to be largest for AQP5, and just slightly less for AQP6 and AQP1, such that these three seem to be the best conductors of CO_2_. Substantial ΔpH_S_ values are also exhibited by AQP0, AQP4-M23 (a variant of AQP4-M1, which forms orthogonal arrays of tetramers in membranes), AQP9, and perhaps AQP8. Little or no CO_2_ permeability is contributed by AQP2, AQP3, AQP4-M1, and AQP7. Functionally, it is notable that high permeabilities for CO_2_ seem to be exhibited by the most widespread isoform AQP1, occurring for example in red cells and the kidney, by the AQP4-M23, which is present in high density in the astrocytic endfeet at the blood-brain barrier, by AQP5, which is strongly expressed in alveolar type I pneumocytes and thus may facilitate diffusion of CO_2_ across the alveolar-capillary barrier, and finally by AQP6, which is present and colocalized with H^+^-ATPase in the intracellular vesicle membranes in the renal collecting duct epithelia. According to the authors' speculation, AQP6 may serve there to mediate CO_2_ exit from the vesicle lumen, after the latter has actively taken up H^+^, followed by its reaction with HCO^−^_3_ to form CO_2_ inside the vesicle. Recently, Geyer et al. (2013b) have shown with their surface pH transient technique that in addition to RhAG the tissue Rh proteins RhBG and RhCG serve as pathways for CO_2_.

In the following second part of this chapter we will present some work that has come to the conclusion that either **CO_2_ channels do not exist or are functionally insignificant:**

Yang et al. (2000) studied the question of the function of AQP1 as a potential CO_2_ channel in a variety of systems, and reached a negative answer for all of them. They studied liposomes into which AQP1 was reconstituted, examined them by stopped-flow spectrophotometry, obtained the very low *P*_CO_2__ as already discussed above and observed no effect of aquaporin-1 incorporation. Since the low *P*_CO_2__ suggests a major unstirred layer or mixing problem, as discussed, it appears possible that an action of AQP1 may have been obscured by the dominating effect of unstirred layers. The same holds for the authors' determination of *P*_CO_2__ of red cells from normal and AQP1-ko mice, which was also done by stopped-flow. Their values of 0.012 and 0.011 cm/s are more than 10 times lower than the values obtained by ^18^O mass spectrometry (Endeward et al., 2006a, 2008) and also than earlier rough estimates of red cell *P*_CO_2__ by Roughton (1959) and Forster (1969), yielding 0.15 and 0.58 cm/s, respectively. Again, it appears quite possible that Yang's low values are due to an unstirred layer or mixing problem in the stopped-flow apparatus, which would render changes in true membrane *P*_CO_2__ insignificant in comparison to a predominant diffusion resistance in the extracellular solution. A third line of evidence presented by Yang et al. (2000) consists of an experiment with anesthetized artificially ventilated mice. These animals were subjected to a step decrease in inspired *p*_CO_2__ from 35 to 0 mmHg. The absolute value and kinetics of the ensuing decrease in arterial *p*_CO_2__ was then followed in normal and AQP1-ko mice, and no differences in either *p*_CO_2__ amplitude or kinetics were observed. To appreciate the implications of this result, one has to recall the steps that could be crucial for the process of CO_2_ equilibration between blood and red cells. Firstly, red blood cells are fundamentally involved in this equilibration possess by chemical reactions and transport processes, and they possess AQP1 in their membrane. However, as discussed by Endeward et al. (2008), the mere lack of AQP1 in the red cell membrane (with RhAG still present) reduces *P*_CO_2__ to about 0.07 cm/s, and these authors showed that this value under resting conditions still allows complete CO_2_ equilibration of the red cell within the capillary transit time. Thus, red cells of AQP1 null mice are not expected to behave functionally differently from normal mice in a gas exchange experiment under resting conditions. The second potentially crucial step is the transfer of CO_2_ across the alveolar-capillary barrier. Among the elements of this barrier, only the endothelial membranes are candidates, as they express AQP1, while the alveolar epithelium does not (Verkman, 1998; Matsuzaki et al., 2009). The latter, as mentioned above, exhibits a strong expression of AQP5. We will analyse the chances of observing an effect of the AQP1 deficiency in the alveolar-capillary barrier in the experiment of Yang et al. (2000) by using the classical concept of the lung diffusing capacity that goes back to Roughton and Forster (1957). Accordingly, we separate the resistance to CO_2_ transfer from lung to red cell into a “diffusion, or membrane” component and a “chemical reaction” component. This is expressed by 1/*D*_*L*_ = 1/*D*_*L,M*_ + 1/θ*Vc*, where D_L_ is the overall diffusing capacity of the lung, the reciprocal of *D*_*L, M*_ is the diffusion or “membrane” resistance comprising that of the alveolar-capillary barrier, θ is the chemical reaction rate in the red cell, and *Vc* the volume of the lung capillary blood. In other words, 1/θ*Vc* is the resistance to CO_2_ transfer provided by the chemical reactions of CO_2_ and associated erythrocytic transport processes. The comparatively small contribution of the diffusion resistance across the alveolar-capillary barrier becomes manifest in the minimum estimate for *D*_*L, M*_, 1100 ml/mmHg/min, which compares with the overall diffusing capacity of the lung *D*_*L*_ of 180 ml/mmHg/min (Schuster, 1987). Thus, *D*_*L, M*_ makes up at most 1/6 of the total resistance for CO_2_ exchange between lung and red cells. In addition, the endothelial membranes make up only a fraction of the diffusion resistance of the alveolar-capillary barrier, 1/*D*_*L, M*_. We conclude from this that, in order to become noticeable in measurements of CO_2_ exchange between blood and alveolar gas, loss of AQP1 from the endothelial membranes would have to cause a dramatic increase in the diffusion resistance of these membranes. Such an enormous effect of AQP1 seems not very likely. The same conclusion has been reached by Swenson et al. (2002), who attempted to inhibit pulmonary AQP by Zn^++^ and, seeing no effect on CO_2_ exchange between diluted blood (10% hematocrit) or red cell-free lung perfusate and alveolar gas, estimated that the alveolar-capillary membrane diffusing capacity D_*L, M*_ would have to be reduced by >90% to lower CO_2_ exchange by 10%. These arguments also apply to the observation of Fang et al. (2002), who in mice lacking AQP1 and/or AQP5 saw no difference to normal mice when they studied the exchange of CO_2_ between fluid-filled alveoli and lung-vessel perfusates. In that experiment, although of great interest because it studies lungs without AQP1 as well as AQP5, it appears likely that the large unstirred fluid zones in the alveoli will drastically decrease apparent *D*_*L, M*_. Therefore, these unstirred fluid zones will probably dominate the process of CO_2_ exchange rather than the permeability of the membranes of the alveolar-capillary barrier. We conclude that the CO_2_ exchange experiments conducted in lungs do not seem to be quite conclusive evidence against the function of AQP1 (or AQP5) as CO_2_ channels, although the quantitative physiological role of these channels in gas exchange in the lung clearly remains debatable and should be studied further.

A short note on the role of AQP1 in the kidney: Fang et al. (2002) have isolated proximal tubular vesicles, which are known to strongly express AQP1, and measured their CO_2_ permeability by the stopped-flow technique. As for the other vesicles studied by this group and already discussed above, the *P*_CO_2__ they obtained was very low, 0.0035 cm/s, and it was identical between vesicles prepared from normal and AQP1-deficient mice. So the reservations against the stopped-flow technique explained above also apply to these experiments. On the other hand, Boron (2010) presents a preliminary report by Zhou et al. (2006), describing experimental work on intact proximal tubules. They come to the conclusion that 60% of the CO_2_ absorbed in the proximal tubule seems to be due to AQP1.

Missner et al. (2008) in a more recent paper presented essentially two arguments against a functional role of CO_2_ channels in membranes. Firstly, they reported, as mentioned above, a CO_2_ permeability of 3.2 cm/s in planar lipid bilayers, measured at an extremely alkaline pH of 9.6. At this alkaline pH, in the presence of CO_2_, HCO^−^_3_ and CO^−^_3_-, CO_2_ diffusion within the considerable unstirred layer of >100 μm was accelerated due to a facilitated diffusion mechanism that had also been utilized for such measurements by Gutknecht et al. (1977). This is expected to result, as the authors illustrate theoretically, in an only modest *p*_CO_2__ gradient in the unstirred layer adjacent to the planar lipid bilayer, but in a significant *p*_CO_2__ drop across the bilayer. This drop in *p*_CO_2__ is an expression of the transmembrane resistance for CO_2_. It also is the basis of the figure of 3.2 cm/s they obtain for *P*_CO_2__ from pH profile measurements in the front unstirred layer. The experimental procedure *per se* was similar to the one described above for the experiments of Zocher et al. (2012). We point out, however, the limitations of the fitting procedure used by Missner et al. (2008) to derive *P*_CO_2__ values, as already voiced in chapter I of this article. Missner et al. (2008) take the *P*_CO_2__ value of 3.2 cm/s to be representative for cell membranes in general, and go on to argue that with an unstirred layer (USL) of 1 μm as one often has around cells, the resistance of the USL vs. that of the membrane will be:

(7a) $$\begin{array}{l} d_{\text{USL}}/D_{{\text{CO}}_{\text{2}}}+1/P_{{\text{CO}}_{\text{2}}}=\text{total apparent membrane diffusion}\\ \text{ resistance, or, inserting numbers:} \end{array}$$

(7b) $$\begin{array}{l} 1\cdot 10^{-4}\text{cm}/1.8\cdot 10^{-5}{\text{cm}}^{\text{2}}\text{/s}+1/3.2\text{cm/s}\\ =5.6\text{s/cm}+0.3\text{s/cm}=5.9\text{s/cm}. \end{array}$$

This shows, according to the authors, that the resistance to CO_2_ diffusion through a cell membrane will always be dominated by the unstirred layer effect. Therefore, they conclude, a CO_2_ channel in the cell membrane can never be of functional significance. We note that the basis of this argument rests on the assumption that the *P*_CO_2__ of the cell is identical to the *P*_CO_2__ of a lipid bilayer. This is in fundamental contrast to the determinations of red cell *P*_CO_2__ (Endeward et al., 2006a, 2008), where even in the presence of CO_2_ channels values of 0.15 cm/s were found, and in their absence an estimate of ~0.01 cm/s was obtained. It is also in contrast to the findings of Itel et al. (2012) who report a dramatic decrease of bilayer *P*_CO_2__ over 2–3 orders of magnitude with increasing cholesterol content, and it is in contrast to the *P*_CO_2__ of 0.01 cm/s observed for cells in culture (Itel et al., 2012) as well as to earlier measurements of a *P*_CO_2__ of 0.001 cm/s for the apical membrane of colon epithelium (Endeward and Gros, 2005). The calculation of Equation (7a), when performed for a *P*_CO_2__ of 0.01 cm/s, leads to a 25 times greater diffusion resistance of the membrane in comparison to that of the 1 μm thick unstirred layer. Thus, the available experimental *P*_CO_2__ values of cell membranes suggest that protein CO_2_ channels can indeed be physiologically significant.

The second argument presented by Missner et al. (2008) against the relevance of CO_2_ channels in biological membranes comes from measurements of the flux of CO_2_ across monolayers of MDCK cells. They study native MDCK cell layers in comparison to MDCK cells expressing AQP1 and substantiate the presence of AQP1 by immunocytochemistry and by water flux measurements. In terms of CO_2_ flux, they find no difference between MDCK cells with and without AQP1. In order to appreciate this latter result, one has to analyse the experimental situation employed by Missner et al. (2008). We do this in the following along the lines already published by Endeward and Gros (2009). The cells are grown on semipermeable supports by Transwell (Corning), whose exact type is not given, but which may have been 50 μm thick. The flux measurement then was done across the sum of the MDCK monolayer, which may have been 10 μm thick, plus the 50 μm support, plus an unstirred layer on the back side, behind the support, of the thickness demonstrated by the authors for the front side to be at least 130 μm. This adds up to a total water phase of at least 190 μm in thickness, across which the flux of CO_2_ occurred. We ignore the front side unstirred layer, because that was controlled by the microelectrode measurement and gave the authors the precise front-side surface pH value. If we now perform, as above in Equation (7), an analysis of the diffusion resistances of the overall water phase and of the membranes, we can write:

(8) $$\begin{array}{l} d_{\text{w}}/D_{{\text{CO}}_{\text{2}}}+1/P_{{\text{CO}}_{\text{2},\text{b}}}+1/P_{{\text{CO}}_{\text{2},\text{a}}}=\text{total diffusion resistance}\\ \text{ of the combined layer,} \end{array}$$

where d_w_ is the overall thickness of the combined layers, D_CO_2__ the CO_2_ diffusion coefficient in water (taken to approximate diffusion in all three components of the layer), *P*_CO_2, b__ the CO_2_ permeability of the basal MDCK membrane, and *P*_CO_2, a__ the CO_2_ permeability of the apical membrane. To insert numbers, let us first use Missner's assumed *P*_CO_2__ for cells, 3.2 cm/s:

(8a) $$\begin{array}{l} 190\cdot 10^{-4}\text{cm}/1.5\cdot 10^{-5}{\text{cm}}^{\text{2}}\text{/s}+1/3.2\text{cm/s}+1/3.2\text{cm/s}\\ =1270\text{s/cm}+0.3\text{s/cm}+0.3\text{s/cm}=1271\text{s/cm} \end{array}$$

This would imply that the membrane resistance makes up <0.05% of the total resistance, and no change in membrane *P*_CO_2__ by a factor even of 10 would ever be detectable. Thus, if we accept the logic of the paper by Missner et al. (2008), an aquaporin effect on membrane permeability could never have been measurable. Thus, the conclusion that AQP1 does not increase membrane CO_2_ permeability is not substantiated by this experiment.

Instead of *P*_CO_2__ = 3.2 cm/s, we can tentatively insert into Equation (8) the value determined by Itel et al. (2012) for isolated MDCK cells, *P*_CO_2__ = 0.017 cm/s:

(8b) 1270s/cm+1/0.017s/cm+1/0.017s/cm=1390s/cm

Even with this much lower membrane permeability, the two membranes contribute <10% of the total diffusion resistance. An increase in *P*_CO_2__ due to AQP1 expression by ~50% as reported by Itel et al. (2012), would necessitate being able to measure the difference between total diffusion resistances of 1390 s/cm and 1350 s/cm, i.e., a difference of less than 3%. In view of the precision of these measurements—and of any other conceivable experimental technique—it seems impossible to detect such an effect of AQP1 on membrane *P*_CO_2__. No matter which membrane *P*_CO_2__ we accept, the experiment used by Missner et al. (2008) seems to be unsuitable to observe AQP effects on *P*_CO_2__. The decisive flaw in this setup is the large overall thickness of the diffusion layer.

**Figure 7:**
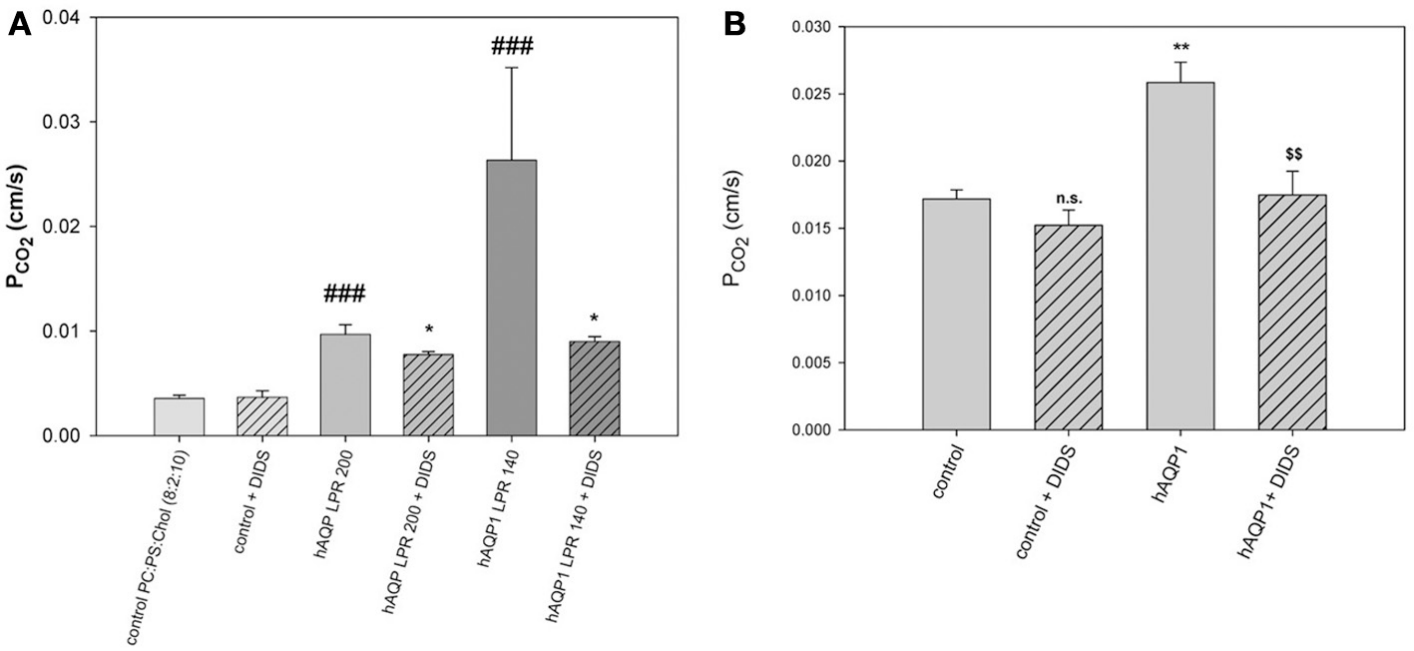
**Effects of AQP1 incorporation into lipid vesicles and into MDCK cell membranes. (A)** Phospholipid vesicles of phosphatidylcholine:phosphatidylserine 8:2, with 50 mol% cholesterol, with and without incorporation of human AQP1. LPR is lipid-to-protein ratio. At the highest protein incorporation, LPR 140, the *P*_CO_2__ as measured by ^18^O exchange mass spectrometry is 9-fold increased over the control (leftmost column). DIDS reduces the AQP1-mediated CO_2_ conductances, but not the *P*_CO_2__ of control vesicles. ^###^Significantly different from AQP1-free control vesicles, *P* < 0.01; ^*^significant effect of DIDS on vesicles with the same LPR, *P* < 0.05. **(B)** Expression of human AQP1 in MDCK cells increases *P*_CO_2__ significantly (^**^P < 0.02). DIDS reduces the hAQP1-mediated increase in *P*_CO_2__ (^$$^*P* < 0.02), but does not affect *P*_CO_2__ of control cells. Reproduced from Itel et al. (2012), with permission.

Summarizing this chapter with the pros and contras on the existence of CO_2_ channels, we feel that overwhelming evidence has been accumulated by now that aquaporins and Rhesus protein indeed act as CO_2_ channels, and several lines of evidence proposed against CO_2_ channels are subject to some critique. Whether these channels are physiologically important or not, indeed depends crucially on the question what the “intrinsic” CO_2_ permeability of a cell membrane is. If it is very high, channels do not make sense, if it is low, they do. A small paper by representatives of the opposing views on this topic, Boron, Endeward, Gros, and Pohl, based on their discussion at a meeting in Strobl (Austria), was published (Boron et al., 2011) and summarizes this state of the discussion. After this meeting, the paper by Itel et al. (2012) has been published that shows that many membranes, due to their cholesterol content, have rather low intrinsic CO_2_ permeabilities and thus channels do make sense. This paper demonstrates this by (a) incorporating AQP1 into artificial phospholipid vesicles containing 50% cholesterol, which increases the vesicles' *P*_CO_2__ up to 9-fold (Figure 7A), and (b) by showing that expression of AQP1 in MDCK cells, which possess ~40% cholesterol, significantly increases membrane CO_2_ permeability by ~50% (Figure 7B). Both experiments clearly illustrate that AQP1 acts as a functionally relevant CO_2_ channel, if the “intrinsic” membrane permeabilities for CO_2_ are sufficiently low, ~0.003 cm/s in the case of vesicles and 0.017 cm/s in the case of the MDCK cells. As most cells have cholesterol contents of around 40%, most cells are expected to exhibit an intrinsic CO_2_ permeability in the order of 0.01 cm/s, and thus their CO_2_ permeability will be significantly enhanced by the incorporation of a protein gas channel. It appears therefore at this stage that nature has two options to achieve a high CO_2_ permeability of a cell membrane, either by reducing its content of cholesterol, which will be at the expense of a reduced barrier function to other molecules and of a reduced mechanical stability, or by maintaining a high cholesterol content and raising CO_2_ permeability by incorporating protein gas channels. It will be of interest in the future to find more biological examples of these two options. A third option is of course to combine high cholesterol content with the absence of gas channels, which will give “gas-impermeable” membranes as discussed above.

## A brief view on other gases of physiological interest

Direct determinations of cellular gas permeabilities for the gases listed in Table 1 other than CO_2_ are sparse. Kutchai (1975) concluded that the permeabilities of red cell membranes for O_2_ and CO are high enough in order to constitute no significant diffusion resistance during O_2_ and CO uptake by these cells, but did not come up with numbers for these permeabilities. Honig et al. (1992) observed by oxymyoglobin microspectrophotometry a shallow *p*_O_2__ gradient inside skeletal muscle cells and concluded that a steep gradient of *p*_O_2__ must occur between the surface of the red cell and the sarcolemma. In this phenomenon, however, the authors implied neither the red cell membrane nor the sarcolemma as sites of major diffusion resistance to O_2_, although this would have been conceivable, but rather attributed this resistance to the interstitial myoglobin-free space. However, in a later paper Voter and Gayeski (1995) found that the spatial resolution in the earlier experiments was considerably poorer than previously thought, so the intracellular *p*_O_2__ gradients were in fact greater, reducing the role of the space between red cell and muscle cell membrane as a diffusion resistance for O_2_. In the present context, these data do not give an indication for a significant role of the two membranes as diffusion resistances toward O_2_. For lipid vesicles, Widomska et al. (2007) used the saturation-recovery EPR technique to determine the oxygen permeability coefficient. They reported extremely high *P*_O_2__ values of 51, 50, and 157 cm/s for lipid bilayers at 37°C made from lipids extracted from membranes of the lens, from a 1-palmitoyl-2-oleoylphosphatidylcholine/cholesterol mixture, and from pure 1-palmitoyl-2-oleoylphosphatidylcholine, respectively. The same group has reported measurements with this technique in the Chinese hamster ovary plasma membrane, finding a *P*_O_2__ of 42 cm/s at 37°C (Subczynski et al., 1992). These values, if true for cell membranes in general, would be far beyond constituting a barrier to O_2_ supply to cells, and they would deviate drastically from the value predicted in Table 1 from the measured *P*_CO_2__ and the gas partition coefficients. If the very high intramembrane diffusivity of O_2_ arising from these high *P*_O_2__ values were true for O_2_ and perhaps other gases such as NO, CO, and N_2_ as well, the implication would be that even in a pure phospholipid membrane, D_M_ for CO_2_ would be exceptionally low among these gases. In sharp contrast to these EPR measurements, Ivanov et al. (2004) reported for surface lipid monolayers oxygen permeabilities between 10^−4^ and 10^−5^ cm/s, i.e., 6–7 orders of magnitude lower than the values derived from EPR. As a consequence, they postulated the existence of O_2_ channels in biological membranes. Indeed, Wang et al. (2007) have shown by molecular dynamics simulations that AQP1 can serve as a conduit for O_2_. The group of Echevarría et al. (2007) has also postulated the existence of oxygen channels, not from direct O_2_ flux measurements, but based on several lines of indirect evidence. e.g., they report (a) that overexpression of AQP1 results in an accelerated upregulation of HIF2α after exposure to hypoxia of PC12 cells, (b) that knockdown of AQP1 in endothelial cells induces hypoxia-inducible genes, and finally (c) they observe that rat lungs under systemic hypoxia up-regulate the expression of AQP1. One might ask, though, whether some of these effects could not be related to the function of AQP1 as a water channel.

A somewhat similar situation exists with respect to NO. Sakai et al. (2008) studied the uptake of NO by hemoglobin-loaded artificial vesicles using stopped-flow spectrophotometry. With low intravesicular hemoglobin concentrations and small vesicle sizes, the rate of combination of NO with deoxyhemoglobin was identical to the reaction rate with deoxyhemoglobin in solution. The authors concluded that no resistance of the vesicle membrane was apparent. With increasing vesicle size a retardation of the apparent association rate constant became apparent, which they interpreted as being due to an increasing role of intravesicular gas diffusion. This latter effect was not visible when they measured the uptake of CO by these vesicles, a fact they attributed to the much slower reaction kinetics of the CO-hemoglobin association. Again, in the case of NO, molecular dynamics simulations predict that AQP4 is an especially good conductor of both NO and O_2_ (Wang and Tajkhorshid, 2010), which would not make sense physiologically if the membrane permeabilities were generally immeasurably high. Along this line, in the case of NO a substantial body of evidence has been compiled from AQP1-transfected cells in culture, from AQP1 reconstituted into lipid vesicles, and from physiological measurements of the AQP1-dependence of the physiological actions of NO, all indicating that AQP1 is essential in getting NO across cell membranes from its site of production to its target (Herrera et al., 2006; Herrera and Garvin, 2007).

In conclusion, the field of O_2_ and NO permeation across biological membranes is highly controversial and drastically contradictory results have been reported. This area clearly awaits further studies of the mechanisms by which these gases cross membranes.

## A discussion of the suitability of the ^18^O exchange mass spectrometric technique to measure the fast process of CO_2_ permeation

We have discussed above that CO_2_ exchange with lipid vesicles of a diameter of 150 nm may not be measurable by rapid reaction stopped-flow techniques, which have dead-times of a few milliseconds, provided the mixing efficiency of the apparatus is very good. This is so because the process is much faster than the dead time, if intravesicular CO_2_ hydration velocity is not limiting and if there is no marked diffusion resistance offered by the vesicle membrane. How can under these premises the mass spectrometric ^18^O technique overcome the problem of the extreme rapidity of the processes of CO_2_ permeation? In this chapter we will first summarize the principle of the ^18^O exchange technique, which has already been described before in great detail, then address the above question how a mass spectrometric technique, whose response time is 3 s (Endeward and Gros, 2005) and which records its signal on a time scale of several hundreds of seconds, can be able to describe the extremely fast process of CO_2_ exchange across cells and vesicles. Next, we will demonstrate the sensitivity of the calculated permeability values for CO_2_ to other parameters that enter into the calculation, then illustrate quality and reliability of the fitting procedure that needs to be employed, because an analytical solution of the system of differential equations describing the ^18^O exchange process is not available, and finally discuss experimental data on the effect of unstirred layers in the mass spectrometric *P*_CO_2__ measurements of red cells.

### Principle of the method

**Figure 8:**
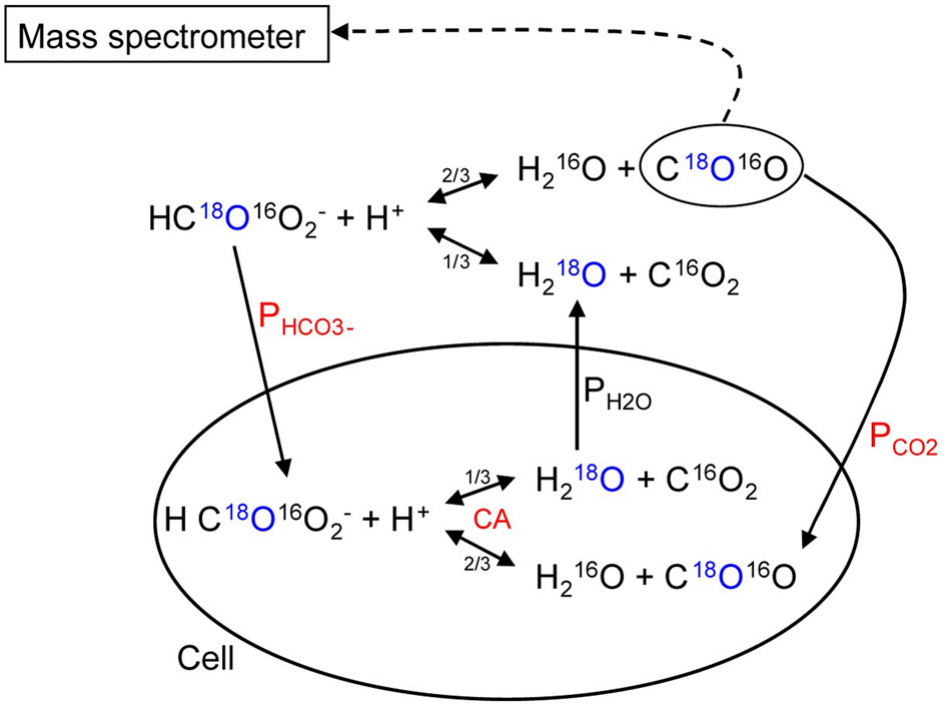
**Schematic representation of the processes occurring when carbonic anhydrase-containing cells are added to ^18^O-labeled CO_2_-HCO^−^_3_ solution**. The labeled O is marked in blue, the most critical parameters determining the time course of the process are labeled in red. Single arrows indicate transport processes, double arrows reaction processes.

**Figure 9:**
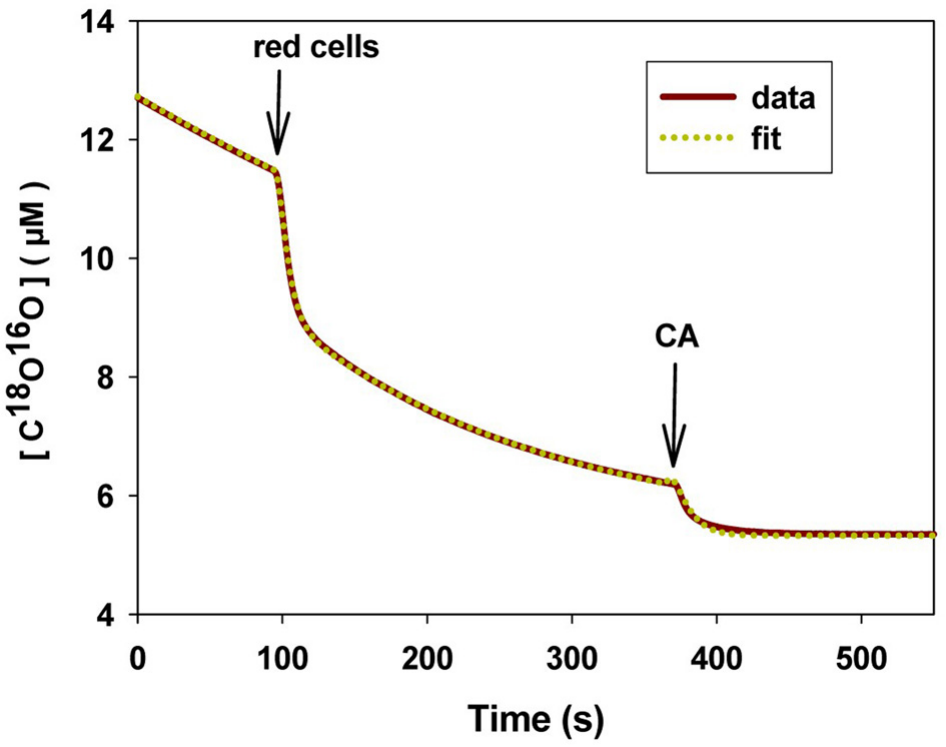
**Original mass spectrometric recording of an experiment with human red cells**. The concentration of C^18^O^16^O is plotted vs. time. First arrow point of addition of red cells, second arrow point of addition of CA in excess to rapidly establish final isotopic equilibrium. Solid curve (red): mass spectrometric recording; dotted curve (yellow): calculated curve using the best-fit values of *P*_CO_2__ and *P*_HCO^−^_3__. 37C.

We will briefly summarize the mass spectrometric method to measure *P*_CO_2__ of cells and vesicles, which has first been described in detail by Wunder and Gros (1997, 1998) and Wunder et al. (1998), and later in its final form by Endeward and Gros (2005). All technical details are described in these papers. The underlying principle is that used by Mills and Urey (1940) to measure the kinetics of the CO_2_ hydration-dehydration reaction by using ^18^O-labeled CO_2_ and HCO^−^_3_ and observing the very slow process of the disappearance of the ^18^O from the CO_2_/HCO^−^_3_ pool into the comparatively huge H_2_O pool. Much later, Itada and Forster (1977) developed an ingenious gas inlet system for the mass spectrometer and used this method to observe the effect of the addition of red cells to the solution of labeled CO_2_/HCO^−^_3_upon the time course of ^18^O loss from CO_2_. They calculated from this the intraerythrocytic carbonic anhydrase activity and the red cells' bicarbonate permeability. Wunder and Gros (1997, 1998) and Wunder et al. (1998) became aware of the fact that this time course also contains information on the CO_2_ permeability of the red cell membrane and they developed the theory describing this process, which allowed them to derive *P*_CO_2__ values from the mass spectrometric recordings. Because some refinements of the theory were added later on, we will in the following refer to the theory in the form described by Endeward and Gros (2005). NaHC^18^O^16^O_2_ is dissolved in a solution in the reaction chamber connected to the mass spectrometer via the previously described gas inlet system (Wunder and Gros, 1998). The ^18^O-labeled bicarbonate rapidly establishes chemical equilibrium with ^18^O-labeled CO_2_, and from then on chemical equilibrium exists in the system, and the entire remainder of the experiment occurs under conditions of perfect chemical equilibrium, including constant values of pH and total *p*_CO_2__. However, the system at this point is far from isotopic equilibrium, which is approached very slowly by the mechanism indicated in the upper half of Figure 8. Each time the labeled bicarbonate reacts to give water and CO_2_, there is a 1/3 chance that the ^18^O is lost from the CO_2_-HCO^−^_3_ pool into the water pool. Since the latter is 55.5 M/20 · 10^−3^M ≈ 3000 times greater than the former, eventually almost all the ^18^O will be lost into the water. This causes a slow spontaneous decay of C^18^O^16^O, the quantity measured by the mass spectrometer via the gas inlet system. This decay is seen in the first part of the recording of Figure 9. The slope of this first part is what Mills and Urey (1940) used to determine the CO_2_ hydration velocity constant. Both, Figures 8, 9 show that the situation changes dramatically, when red cells (or other carbonic anhydrase-containing cells or vesicles) are added. This is followed by a (rapid) influx of labeled CO_2_ into the red cells, where it is fed into the same reaction processes as shown for the extracellular space in the upper half of Figure 8. More slowly than C^18^O^16^O, labeled bicarbonate then also enters the red cells and is fed into these reactions. These two processes can be assigned—although not exclusively—to the two phases seen in the mass spectrometric recording in Figure 9 after red cell addition: the first rapid phase is dominated by the CO_2_ influx (besides by the intracellular carbonic anhydrase (CA) activity), and the second slower phase contains most of the information about the influx of HCO^−^_3_. The same reaction processes that occur in the extracellular space occur in the intracellular space 20,000 times faster due to the CA, and for this reason almost the entire reaction process now takes place intracellularly. As evident from the scheme of Figure 8, the intracellular reaction process depends on CO_2_ and HCO^−^_3_ moving into the cells, a process which is controlled by the permeabilities of the membrane for CO_2_, *P*_CO_2__, and for HCO^−^_3_, *P*_HCO^−^_3__. All the reaction and diffusion processes of Figure 8 are described by a set of six differential equations (Endeward and Gros, 2005), and their numerical solution is used to determine by a fitting procedure the best-fit values of these two parameters. It should be noted that the intracellular CA activity, which is also very important for the kinetics of the ^18^O exchange process, is determined independently from cell lysates under intracellular conditions and is not obtained from the fitting procedure. Determining CA activity in this way requires ensuring that two important parameters influencing specific CA activity equal those present inside cells, i.e., pH and [Cl^−^], and it presupposes that the CA activity measured in diluted cell lysate can safely be extrapolated to the intracellular CA concentration. This latter assumption has previously been validated by the work of Donaldson and Quinn (1974). The perfect superimposition of the original mass spectrometric recording in Figure 9 (solid red curve) and the theoretical curve (dotted yellow) calculated with the best-fit values of *P*_CO_2__and *P*_HCO^−^_3__ is achieved for almost all experiments. It illustrates that the theory provides an excellent description of the process of Figure 8.

### How can the ^18^O exchange mass spectrometric method measure processes that occur within some milliseconds?

The difference between the kinetics of cellular CO_2_ exchanges and the kinetics of the decay of the ^18^O-labeled CO_2_ reflecting these exchanges is huge. For example, let us consider the CO_2_ uptake process by human red cells. This process is so fast that it is tricky to measure it by the stopped-flow technique, for the reasons already discussed, dead time of a few milliseconds, possible imperfect mixing, and the special problem encountered when mixing cell or vesicle suspensions in this apparatus. For this reason, stopped-flow is not an ideal technique to measure the uptake of gases by cells, and we will not refer to such measurements here. Holland and Forster (1975) measured the uptake of CO_2_ by human red cells with a rapid reaction continuous flow apparatus, which does not have this problem, because the solutions with the cells continue to flow during observation. The authors determined a half-time of this process of 13 ms, which we consider fairly reliable for the reasons just given. Let us compare this number with the kinetics of the uptake of C^18^O^16^O by human red cells, as it is reflected in the extracellular decay of this species and illustrated in Figure 9. Using the point of red cell addition, and the final isotopic equilibrium reached after the addition of excess free CA at the end of the experiment, we can estimate the half-time of the overall process to be ~140 s, which is more than 10,000 times the half-time of net uptake of CO_2_. What is the mechanism of this enormously slow representation of a very fast process in the C^18^O^16^O decay signal? There are two major mechanisms contributing to this, which we shall discuss in the following.

**Figure 10:**
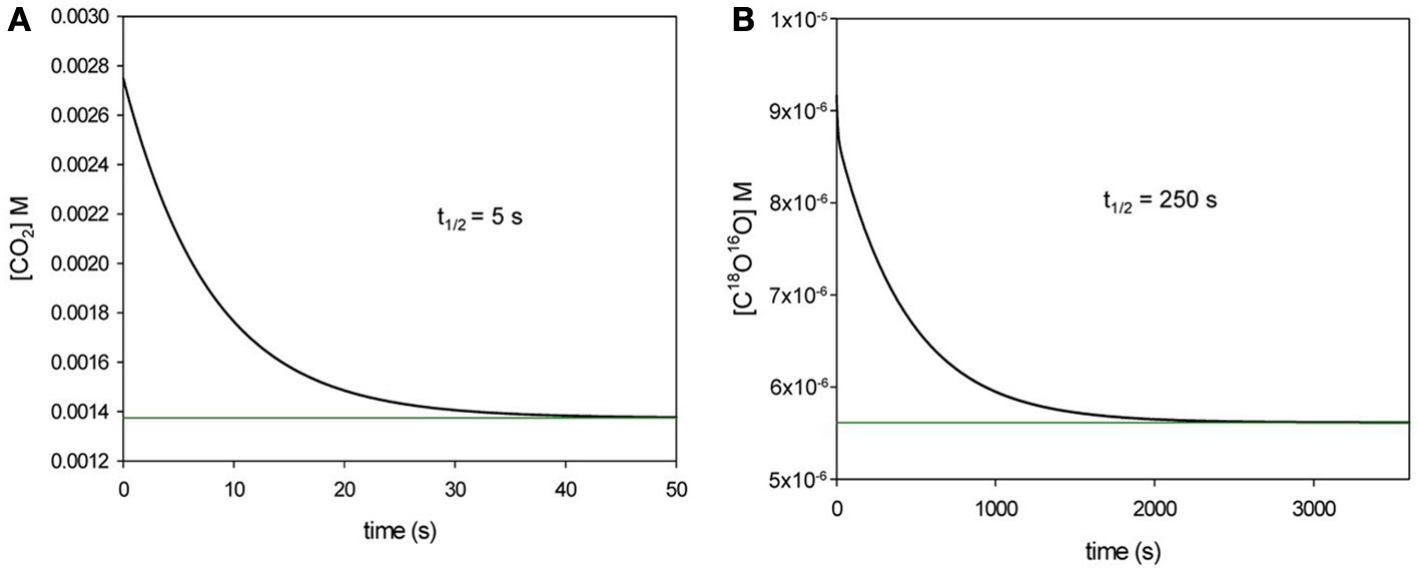
**Comparison of the time courses of the net CO_2_ hydration reaction after a step increase in [CO_2_] (A), and the spontaneous decay of C^18^O^16^O after a step increase in labeled CO_2_ and HCO^−^_3_ being in chemical equilibrium but not in isotopic equilibrium with water (B)**. Calculated for 37°C. Inserts indicate the half-times of the two reactions.

(1) Mills and Urey (1940), who developed the ^18^O exchange technique to measure the kinetics of the uncatalyzed CO_2_ hydration reaction, explained that the disappearance of ^18^O from the labeled CO_2_/HCO^−^_3_ into the water pool will, firstly, be only 1/3 as fast as the net CO_2_ hydration reaction as it is illustrated in Figure 10A, because under chemical, but not isotopic, equilibrium only 1/3 of the reaction steps producing H_2_O and CO_2_ from HCO^−^_3_ will lead to as loss of ^18^O into the water pool (see upper half of Figure 8). Secondly, as most of the ^18^O label is located in HCO^−^_3_ rather than in CO_2_, but each forward and backward reaction step in Figure 8 has to pass through CO_2_, the process will be slowed by the ratio [HCO^−^_3_]/[CO_2_], which for the calculation of Figure 10B we have assumed to be 17, corresponding to a pH of 7.3 at 37°C.

In Figure 10B we have calculated the half time necessary to reach isotopic equilibrium after a step increase in C^18^O^16^O in solution, in the manner in which this is also done for the extracellular space in the equations describing the process of Figure 8. This yields a t_1/2_ of 250 s. In Figure 10A we have calculated the half-time of the net reaction CO_2_ + H_2_O ↔ HCO^−^_3_ + H^+^ at 37°C after a step increase in *p*_CO_2__, giving a t_1/2_ of 5 s. In other words, the decay of C^18^O^16^O occurs 50 times more slowly than the net CO_2_ hydration. The factor 50 results from the product 3 × 17, as predicted by Mills and Urey (1940).

Having accounted for a factor of 50 out of a total slow-down factor of 10,000, we are left with a factor of 10,000/50 = 200.

**Figure 11:**
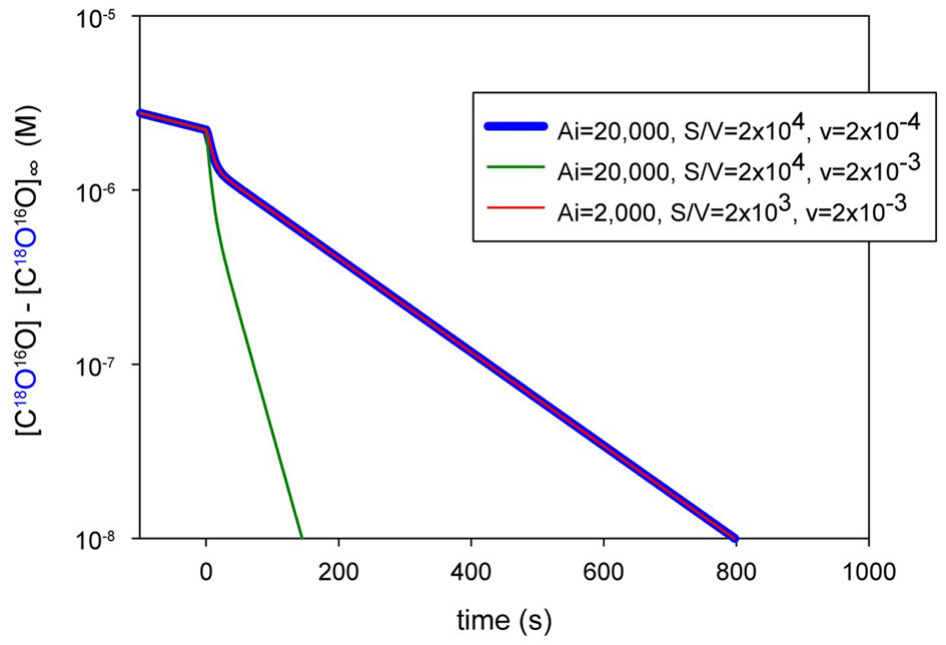
**Calculated mass spectrometric recordings for three different conditions of hematocrit and intracellular activity**. Blue curve: human red cell hematocrit of 0.02% with intracellular activity Ai of 20,000. Green curve: hematocrit of 0.2% with Ai = 20,000. Red curve (superimposed to the blue curve): hematocrit of 0.2% with Ai = 2000 and 10 times smaller surface-to-volume ratio S/V, similar to what is exhibited by MDCK cells etc. Y-axis: actual concentration of C^18^O^16^O at a given time minus its concentration [C^18^O^16^O]∞ after isotopic equilibrium has been reached.

(2) An important point to consider here is that in the mass spectrometric measuring chamber one does not use packed red cells, but highly diluted red cell suspensions. By observing the decay of the extracellular C^18^O^16^O concentration, one does not observe what is going on inside the red cell, but its reflection in the outside solution. Obviously, this signal will vary drastically with the hematocrit in the measuring chamber. However, the optimal hematocrit is not just 1/200 = 0.5%, but even >10 times less (Endeward et al., 2008). The extracellular solution in view of this very low hematocrit contains a huge reservoir of ^18^O, mainly in the form of HC^18^O^16^O^−^_2_. Due to the high intracellular CA activity, the reactions of Figure 8, however, occur almost exclusively inside the cell. This implies that all the label of the extracellular solution has to pass through the very small volume of the red cells on order to end up eventually in the water of the reaction chamber. This situation will slow down greatly the overall process, and is comparable to an RC element with a capacitor of large capacitance (the large pool of ^18^O in the CO_2_/HCO^−^_3_ of the extracellular solution) in conjunction with a resistor (the small red cell volume through which the process of ^18^O decay has to pass) in an electric circuit. Given that the time constant of an RC element is *R* · *C*, an increase in R will slow down the process. Inversely, decreasing R by increasing the hematocrit will accelerate the process. This latter case is illustrated in the curves of Figure 11, which were calculated with our mathematical model describing the scheme of Figure 8. The blue curve illustrates the often used condition of a red cell experiment with a hematocrit in the measuring chamber of 0.02% (with the usual intracellular CA activity of human red cells of 20,000, and a surface-to-volume ratio of these cells of 20,000 cm^−1^). The faster, green curve, which would just be measurable in the mass spectrometer, is calculated for a ten-fold hematocrit of 0.2%. It is apparent that this causes an enormous acceleration of the C^18^O^16^O kinetics, although not quite by a factor of ten, but by a factor of about 5.5. This non-proportional behavior is the reason why, in order to achieve a deceleration of a factor of 200, one needs a greater than 200-fold dilution of packed red cells.

In conclusion, the present mass spectrometric technique is able to measure extremely fast CO_2_ exchanges (i) because it observes the loss of ^18^O from CO_2_+ HCO^−^_3_ into the water pool, rather than observing directly the CO_2_ exchange process, and (ii) because an extreme dilution of the cell suspensions drastically slows down this kinetics. An additional and practically highly useful information can be taken from Figure 11 by noting that the red curve coincides with the blue curve: cells with properties approaching those of MDCK cells that are larger than red cells (with a correspondingly smaller surface-to-volume ratio), but possess, for example, a 10 times lower intracellular CA activity, can be measured equally well, if in compensation the cytocrit is raised ten-fold. This implies that the method is suitable to measure *P*_CO_2__ of cells like MDCK, HEK293 or tsA201 cells with considerably lower intracellular CA activity than red cells (Al-Samir et al., 2013), provided the cytocrit in the measuring chamber is raised correspondingly.

**Figure 12:**
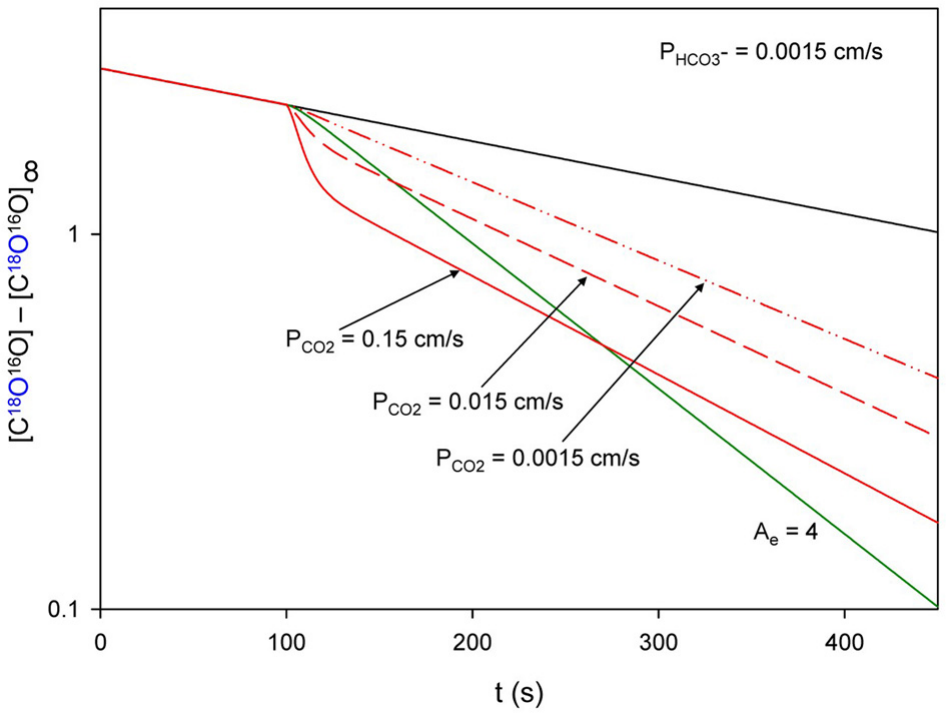
**Calculated time courses of mass spectrometric recordings from human red cells at a hematocrit of 0.02%**. The red curves are calculated for a *P*_CO_2__ of 0.15 cm/s (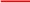), 0.015 cm/s (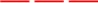) and 0.0015 cm/s (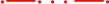). The green line (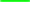) indicates an extracellular CA activity Ae of 4, if free CA is added to the solution in the absence of cells. If the red cells studied in the three red curves were lysed, they would also cause an Ae of 4. Denotation of axes identical to Figure 11.

The effect of using the extremely low hematocrits in these experiments is further visualized in Figure 12. With Ai = 20,000, a hematocrit of 0.02% implies an overall CA activity in the measuring chamber of 20,000 × 0.0002 = 4. The green line in Figure 12 shows the effect of adding an amount of free CA in solution into the chamber that yields a four-fold acceleration of the slope of C^18^O^16^O decay. The red curves have been calculated for a hematocrit of intact red cells of 0.02% and an Ai of 20,000, using different *P*_CO_2__ values of 0.15, 0.015, and 0.0015 cm/s. The three curves are distinctly different, especially in terms of the kinetics and amplitude of their first phases, and thus conspicuously reflect the different *P*_CO_2__ values. The overall CA activity of the red cells studied in the three red curves, however, when averaged over the entire chamber volume, is 4. This activity when present free in solution is indicated by the green line (Ae = 4). It may be seen that the gross overall time course of all three red cell curves is similar to that of the green curve for Ae = 4. We conclude that the extreme dilution of the cells investigated, which this method allows us employ, makes it possible to slow the process of ^18^O exchange sufficiently down to make it measurable by mass spectrometry.

### Sensitivity of the *P*_CO_2__ values estimated from the mass spectrometric records for the parameters used in the theoretical treatment

**Figure 13:**
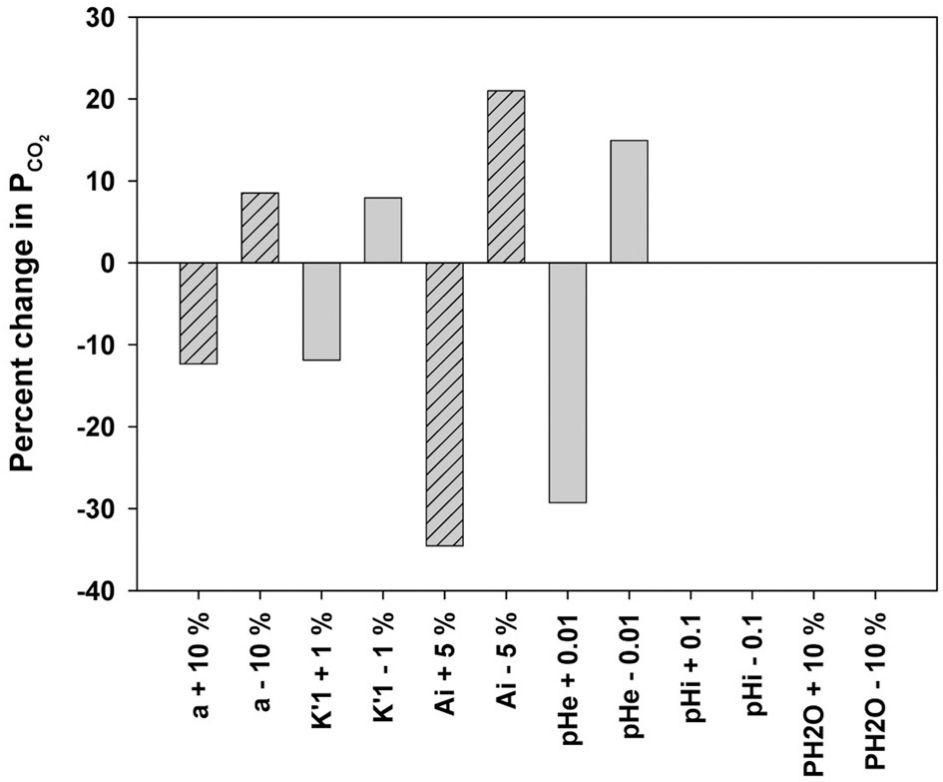
**Sensitivity of the estimated *P*_CO_2__ for variations in the parameters used in the system of equations decribing the processes of Figure 8**. a, Surface-to-volume ratio of cells; K_1_′, first apparent disscociation constant of carbonic acid: K_1_′ = [H^+^] [HCO^−^_3_]/[CO_2_]; Ai, intracellular CA activity; pHe, extracellular pH in measuring chamber; pHi, intracellular pH; *P*_H_2_0_, water permeability of cell membrane. Reproduced from Endeward et al. (2008), with permission.

In addition to the two parameters to be determined, *P*_CO_2__ and *P*_HCO^−^_3__, the parameters given on the x-axis of Figure 13 are used in the set of differential equations (Endeward and Gros, 2005). It is apparent that two very sensitive parameters are the intracellular CA activity, Ai, and the extracellular pH, *p*He, as it prevails in the mass spectrometric measuring chamber. As mentioned, Ai is carefully determined independently. pHe is equally carefully controlled by a pH electrode present in the measuring chamber and always kept precisely at pH 7.40 (Endeward and Gros, 2005; Endeward et al., 2006a). *K*_1_′, the first apparent dissociation constant of carbonic acid, is a precisely known physico-chemical constant. *p*Hi, the intracellular pH, is usually well defined by the *p*He and is entirely uncritical, as is the water permeability of the membrane, *P*_H_2_0_. Even variations of *P*_H_2_0_ with AQP1 expression or lack thereof do not influence the results, because *P*_H_2_0_ is always high enough to warrant a sufficient efflux of labeled water from the cells, a process illustrated Figure 8. An important parameter next to Ai and pHe is the cellular surface-to-volume ratio a, which is determined on each experimental day for all cell types employed. In the case of red cells the volume is derived from hematocrit and cell density, and literature values are used for the surface area. In the case of isolated primary cells or cells isolated from cultures, we determine microscopically cell density and cell diameter and utilize their usually spherical shape to calculate a. In the latter case, the uncertainty is greater than in the case of red cells, but is not expected to exceed 10%, a number which would introduce an error of equal percentage into *P*_CO_2__.

In the context of discussing the influence of Ai on the determination of *P*_CO_2__, it seems appropriate here to add a note on how the ^18^O method behaves when cells of larger size but with much lower intracellular CA activity than red cells, such as MDCK cells, are to be measured. We consider cells with an intracellular CA activity of 500, i.e., 1/40 of that of red cells, which are employed at a suitable cytocrit and possess a true *P*_CO_2__ of 0.01 cm/s. We compare two curves of the kind shown in Figure 9, one fitted perfectly by *P*_CO_2__ = 0.01 cm/s, the other calculated with identical parameters except a value of *P*_CO_2__ of 0.1 cm/s forced upon the calculation. We determine from these curves the concentrations [C^18^O^16^O] shortly after the beginning of the 2nd slower phase, which follows the 1st phase initiated by the addition of cells, and find that these concentrations differ between the two curves by 20%. This is a highly significant difference, which shows that in these cells, similar to the case of red cells (see Figure 1 in Endeward et al., 2008), CO_2_ permeabilities between 0.01 and 0.1 cm/s can be distinguished with great certainty. If we do the analogous calculations for *P*_CO_2__ = 0.1 cm/s and *P*_CO_2__ = 1 cm/s, the difference in [C^18^O^16^O] is 3%, i.e., a distinction between these permeabilities is still possible, but with decreasing reliability. This behavior is similar to that exhibited by red cells. Testing the sensitivity of *P*_CO_2__ (0.01 cm/s) of these MDCK-type cells for changes in Ai, in the manner shown in Figure 13 for red cells, we find that an increase in Ai from 500 to 525 (+5%) causes a decrease in *P*_CO_2__ by 11%, and a decrease in Ai from 500 to 475 (−5%) causes an increase in *P*_CO_2__ by 26%. Thus, for cells of low CA activity the sensitivity of *P*_CO_2__ for errors in Ai is equal to or lower than in the case of red cells as shown in Figure 13. The overall conclusion from these considerations is that the mass spectrometric method works equally well for cells with rather low Ai, e.g., of the type of MDCK cells, as it does for red cells.

### Uniqueness and precision of the permeabilities obtained from the fitting procedure

**Figure 14:**
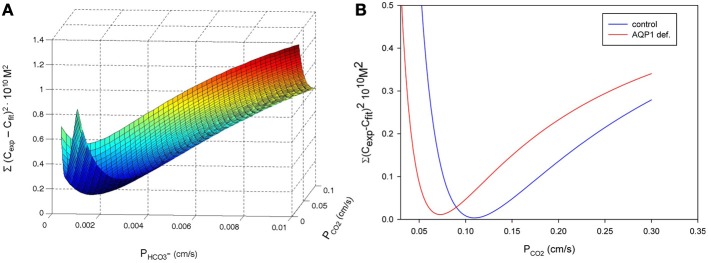
**Quality of the fitting procedure, expressed as sum of squared deviations between experimental and calculated concentrations of C^18^O^16^O**. **(A)** Two-dimensional representation illustrating that a unique minimum of the sum of squares defines *P*_CO_2__ as well as *P*_HCO^−^_3__ for a given experimental record of C^18^O^16^O decay. There are no local minima whatsoever. Reproduced from Al-Samir et al. (2013), with permission. **(B)** Sum of squares for an experimental curve with normal human red blood cells (blue curve) and with AQP1-deficient human red cells (red curve) as a function of *P*_CO_2__. It is apparent that the different CO_2_ permeabilities of the two red cell types are well defined. The precision achieved by the fitting procedure for *P*_CO_2__ is better than 0.005 cm/s.

It is apparent from Figure 14A that the fitting procedure yields highly reliably the values of *P*_CO_2__as well as *P*_HCO^−^_3__ from a given experimental record. This is due to the above-mentioned fact that the curve of C^18^O^16^O decay that evolves after the addition of the cells, exhibits two clearly separated phases. Each of the two permeabilities does not solely determine one of these phases, but *P*_CO_2__ dominates the first rapid phase (together with Ai), while *P*_HCO^−^_3__ plays a minor role, and *P*_HCO^−^_3__ is dominant in the second slower phase, where Ai and *P*_CO_2__ play minor roles. This situation is extremely useful in unambiguously deriving the two permeabilities from the mass spectrometric records, and for the same reason the fitting procedure converges exceptionally well. Figure 14A shows that there is a unique minimum in the sum of squared deviations between experimental and calculated concentrations of labeled CO_2_ for both *P*_CO_2__ and *P*_HCO^−^_3__, and no local minima are present. Figure 14B illustrates for the examples of normal human red cells (blue curve) and of AQP1-deficient human red cells (red curve) how well this method differentiates between the two cell types. Normal red cells in this example have a *P*_CO_2__ of about 0.11 cm/s, and AQP1-deficient red cells of 0.07 cm/s, both values being in the range of values reported by Endeward et al. (2006a). The precision of these determinations is visibly better than 0.005 cm/s.

### Role of unstirred layers in mass spectrometric measurements of red cell CO_2_ permeability

Because the diffusion resistance of cell membranes to CO_2_ is in many cases—as discussed above—small, e.g., compared to that of ions, an additional resistance due to an unstirred layer of water around the cells studied is of importance. It is has therefore often been postulated that CO_2_ permeabilities were underestimated due to the presence of extracellular unstirred layers. Endeward and Gros (2009) have therefore developed an approach allowing them to determine the unstirred layer thickness around red cells in mass spectrometric *P*_CO_2__ determinations.

From the additivity of the diffusion resistances of the membrane and the extracellular unstirred layer it follows that

(9) $$1/P_{{\text{CO}}_{\text{2},\text{app}}}=1/P_{{\text{CO}}_{\text{2},\text{true}}}+\delta /D_{{\text{CO}}_{\text{2}}},$$

where *P*_CO_2, app__ is the measured apparent CO_2_ permeability that includes effects by an unstirred layer, *P*_CO_2, true__ is the true CO_2_ permeability of the membrane, δ is the thickness of the extracellular unstirred layer, and D_CO_2__ is the CO_2_ diffusion coefficient in water that describes CO_2_ diffusion in the unstirred layer.

**Figure 15:**
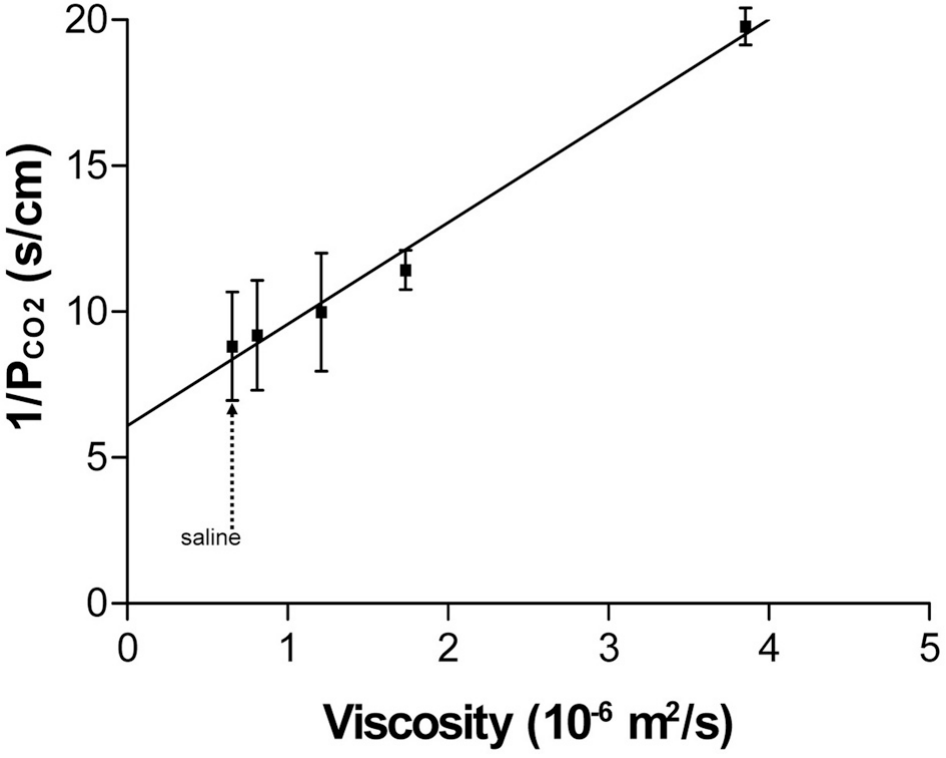
**Plot of the reciprocal of the experimentally determined red cell *P*_CO_2__ (*P*_CO_2, app__) vs. the kinematic viscosity of the dextran solution in which it was measured**. Extrapolation of the linear relation to zero viscosity yields the true membrane *P*_CO_2__ (*P*_CO_2, true__). 37°C. “Saline” indicates the *P*_CO_2__ measured in the absence of dextran. Reproduced from Endeward and Gros (2009), with permission.

It is known from theoretical hydrodynamics that at given other conditions δ increases with increasing solution viscosity ν (Landau and Lifschitz, 1991). To utilize this relationship, Endeward and Gros (2009) suspended human red cells in solutions with dextran concentrations (mol.wt. 60,000) between 0 and 10 g%, whose viscosities varied between 0.7 and about 4 · 10^−6^ m^2^/s. At various dextran concentrations within this range they determined *P*_CO_2, app__ at 37°C, and plotted *P*_CO_2, app__ vs. the viscosity of the dextran solution. The results are shown in Figure 15, which illustrates that a linear relationship between 1/*P*_CO_2, app__ and ν is obtained experimentally. It follows from hydrodynamics (Landau and Lifschitz, 1991) that the unstirred layer must be zero at zero viscosity. Therefore, the extrapolation of the linear regression line in Figure 15 to ν = 0 gives the true membrane CO_2_ permeability *P*_CO_2, true__, which turns out to be 0.16 cm/s. The lowermost experimental data point was obtained in saline (arrow in Figure 15) and had a value of 0.12 cm/s. This means that the unstirred layer reduces *P*_CO_2__ by 25%, which can be considered a minor effect. Inserting numbers into Equation (9), we can write:

$$1/(0.12\text{cm/s})=1/(0.16\text{cm/s})+\delta /(2\cdot 10^{-5}{\text{cm}}^{\text{2}}\text{/s}),$$

and obtain an unstirred layer thickness δ of 4.2 · 10^−5^ cm = 0.42 μm in this case. This again is a very moderate unstirred layer thickness, which was obtained by optimized conventional stirring at maximal speed with a magnetic bar stabilized by being fixed in a Teflon ring. In conclusion, the *P*_CO_2__ values obtained by the mass spectrometric technique are affected by unstirred layers to a minor extent only.

The empirical linear relationship of Figure 15 together with Equation (9) leads to the relation

(10) $$\delta /D_{{\text{CO}}_{\text{2}}}=C\cdot \nu ,$$

where C is a proportionality constant. This implies that

(11) δ~ν,

a prediction derived by Endeward and Gros (2009) from hydrodynamic theory for this experiment, provided the magnetic stirrer exerts a constant force in the cell suspension, independent of viscosity. It should be noted that this latter consideration merely provides a theoretical rationale for the empirical finding of Figure 15.

A topic, although of great interest, has not been discussed here, i.e., the *intracellular* unstirred layer, which is omnipresent in all cellular transport measurements, but is not accessible experimentally. Endeward and Gros (2009) have used a computer model to estimate the size of this layer. For the case of CO_2_ diffusion in red cells they came up with a thickness of this layer of about ¼ to 1/3 of the half-thickness of the erythrocytic disc.

In view of the numerous vesicle experiments discussed in this article, it is of interest to finally add another insight from hydrodynamic theory, which has been presented by Endeward and Gros (2009). When other conditions such as convection are held constant, the thickness of the extracellular unstirred layer is proportional to the square root of the diameter (or “characteristic length”) d of the cell or vesicle studied

(12) δ~√d.

Using δ = 0.42 μm for red cells with a diameter of 7.5 μm, one predicts a δ of 0.06 μm for a vesicle diameter of 150 nm, i.e., the diffusion resistance of the unstirred layer toward CO_2_ will be seven times smaller in vesicles than in red cells. Thus, the estimated vesicular CO_2_ permeability will be corrupted by the unstirred layer to a much lesser extent. Applying an identical consideration on the basis of Equation (12) to MDCK cells (*d* = 10 μm), one comes up with an estimate of δ for MDCK cells of 0.48 μm, just slightly greater than for red cells.

### Conflict of interest statement

The authors declare that the research was conducted in the absence of any commercial or financial relationships that could be construed as a potential conflict of interest.

## References

[B1] Alberts B. Johnson A. Lewis J. Raff M. Roberts K. Walter P. (2002). Molecular Biology of the Cell. 4th Edn New York, NY: Garland Science

[B2] Al-Samir S. Papadopoulos S. Scheibe R. J. Meiβner J. D. Cartron J. P. Sly W. S. (2013). Activity and distribution of intracellular carbonic anhydrase II and their effects on the transport activity of anion exchanger AE1/SLC4A1. J. Physiol. 591, 4963–4982 10.1113/jphysiol.2013.251181 23878365 PMC3810803

[B3] Armstrong A. T. Binkley P. F. Baker P. B. Myerowitz P. D. Leier C. V. (1998). Quantitative investigation of cardiomyocyte hypertrophy and myocardial fibrosis over 6 years after cardiac transplantation. J. Am. Coll. Cardiol. 32, 704–710 10.1016/S0735-1097(98)00296-4 9741515

[B4] Bartels H. Bücherl E. Hertz C. W. Rodewald G. Schwab M. (1959). Lungenfunktionsprüfungen. Berlin-Göttingen-Heidelberg: Springer 10.1007/978-3-642-52983-2

[B5] Battino R. Rettich T. R. Tominaga T. (1984). The solubility of nitrogen and air in liquids. J. Phys. Chem. Ref. Data 13, 563–600 10.1063/1.555713

[B6a] Boron W. F. (2010). Sharpey-Schafer lecture: gas channels. Exp. Physiol. 95, 1107–1130 10.1113/expphysiol.2010.055244 20851859 PMC3003898

[B6] Boron W. F. Endeward V. Gros G. Musa-Aziz R. Pohl P. (2011). Intrinsic CO_2_ permeability of cell membranes and potential biological relevance of CO_2_ channels. Chemphyschem. 12, 1017–1019 10.1002/cphc.201100034 21384488

[B7] Bunce A. S. Hider R. C. (1974). The composition of black lipid membranes formed from egg-yolk lecithin, cholesterol and n-decane. Biochim Biophys. Acta 363, 423–427 10.1016/0005-2736(74)90081-9 4477718

[B8] Crank J. (1957). The mathematics of diffusion. Oxford: Clarendon Press

[B9] Donaldson T. L. Quinn J. A. (1974). Kinetic constants determined from membrane transport measurements: carbonic anhydrase activity at high concentrations. Proc. Natl. Acad. Sci. U.S.A. 71, 4995–4999 10.1073/pnas.71.12.4995 4216027 PMC434026

[B10] Echevarria M. Frindt G. Preston G. M. Milovanovic S. Agre P. Fischbarg J. (1993). Expression of multiple water channel activities in Xenopus oocytes injected with mRNA from rat kidney. J. Gen. Physiol. 101, 827–841 10.1085/jgp.101.6.827 7687270 PMC2216749

[B11] Echevarría M. Muñoz-Cabello A. M. Sánchez-Silva R. Toledo-Aral J. J. López-Barneo J. (2007). Development of cytosolic hypoxia and hypoxia-inducible factor stabilization are facilitated by aquaporin-1 expression. J. Biol. Chem. 282, 30207–30215 10.1074/jbc.M702639200 17673462

[B12] Endeward V. (2012). The rate of the deoxygenation reaction limits myoglobin- and hemoglobin-facilitated O_2_ diffusion in cells. J. Appl. Physiol. 112, 1466–1473 10.1152/japplphysiol.00835.2011 22362405

[B13] Endeward V. Cartron J. P. Ripoche P. Gros G. (2008). RhAG protein of the Rhesus complex is a CO_2_ channel in the human red cell membrane. FASEB J. 22, 64–73 10.1096/fj.07-9097com 17712059

[B14] Endeward V. Gros G. (2005). Low carbon dioxide permeability of the apical epithelial membrane of guinea-pig colon. J. Physiol. 567, 253–265 10.1113/jphysiol.2005.085761 15932894 PMC1474176

[B15] Endeward V. Gros G. (2009). Extra- and intracellular unstirred layer effects in measurements of CO2 diffusion across membranes—a novel approach applied to the mass spectrometric ^18^O technique for red blood cells. J. Physiol. 587, 1153–1167 10.1113/jphysiol.2008.165027 19139045 PMC2674988

[B16] Endeward V. Gros G. Jürgens K. D. (2010). Significance of myoglobin as an oxygen store and oxygen transporter in the intermittently perfused human heart: a model study. Cardiovasc. Res. 87, 22–29 10.1093/cvr/cvq036 20124401

[B17] Endeward V. Musa-Aziz R. Cooper G. J. Chen L. M. Pelletier M. F. Virkki L. V. (2006a). Evidence that aquaporin 1 is a major pathway for CO_2_ transport across the human erythrocyte membrane. FASEB J. 20, 1974–1981 10.1096/fj.04-3300com 17012249

[B18] Endeward V. Cartron J. P. Ripoche P. Gros G. (2006b). Red cell membrane CO_2_ permeability in normal human blood and in blood deficient in various blood groups, and effect of DIDS. Transfus. Clin. Biol. 13, 123–127 10.1016/j.tracli.2006.02.007 16563834

[B19] Fang X. Yang B. Matthay M. A. Verkman A. S. (2002). Evidence against aquaporin-1-dependent CO2 permeability in lung and kidney. J. Physiol. 542, 63–69 10.1113/jphysiol.2001.013813 12096051 PMC2290384

[B20] Finkelstein A. (1976). Water and nonelectrolyte permeability of lipid bilayer membranes. J. Gen. Physiol. 68, 127–135 10.1085/jgp.68.2.127 956767 PMC2228420

[B21] Forster R. E. (1969). The rate of CO_2_ equilibration between red cells and plasma, in CO_2_: Chemical, Biochemical, and Physiological Aspects, eds Forster R. E. Edsall J. T. Otis A. B. Roughton F. J. W. (Washington, DC: National Technical Information Service, NASA SP-188), 275–284

[B22] Forster R. E. Gros G. Lin L. Ono Y. Wunder M. (1998). The effect of 4,4′-diisothiocyanato-stilbene-2,2′-disulfonate on CO2 permeability of the red blood cell membrane. Proc. Natl. Acad. Sci. U.S.A. 95, 15815–15820 10.1073/pnas.95.26.15815 9861053 PMC28127

[B23] Geyer R. R. Musa-Aziz R. Qin X. Boron W. F. (2013a). Relative CO_2_/NH_3_ selectivities of mammalian aquaporins 0–9. Am. J. Physiol. Cell Physiol. 304, C985–C994 10.1152/ajpcell.00033.2013 23485707

[B24a] Geyer R. R. Parker M. D. Toye A. M. Boron W. F. Musa-Aziz R. (2013b). Relative CO_2_/NH_3_ permeabilities of human RhAG, RhBG and RhCG. J. Membr. Biol. [Epub ahead of print]. 10.1007/s00232-013-9593-0 24077989 PMC7684545

[B24] Gros G. Moll W. (1972). The facilitated diffusion of CO_2_ in hemoglobin solutions and phosphate solutions, in Oxygen Affinity of Hemoglobin and Red Cell Acid Base Status, eds Rorth M. Astrup P. (Kopenhagen: Munksgard), 484–492

[B25] Gutknecht J. Bisson M. A. Tosteson F. C. (1977). Diffusion of carbon dioxide through lipid bilayer membranes: effects of carbonic anhydrase, bicarbonate, and unstirred layers. J. Gen. Physiol. 69, 779–794 10.1085/jgp.69.6.779 408462 PMC2215341

[B26] Hasselblatt P. Warth R. Schulz-Baldes A. Greger R. Bleich M. (2000). pH regulation in isolated *in vitro* perfused rat colonic crypts. Pflügers Arch. 441, 118–124 10.1007/s004240000377 11205049

[B27] Herrera M. Garvin J. L. (2007). Novel role of AQP-1 in NO-dependent vasorelaxation. Am. J. Physiol. Renal Physiol. 292, F1443–F1451 10.1152/ajprenal.00353.2006 17229677

[B28] Herrera M. Hong N. J. Garvin J. L. (2006). Aquaporin-1 transports NO across cell membranes. Hypertension 48, 157–164 10.1161/01.HYP.0000223652.29338.77 16682607

[B29] Holland R. A. Forster R. E. 2nd. (1975). Effect of temperature on rate of CO2 uptake by human red cell suspensions. Am. J. Physiol. 228, 1589–1596 236675 10.1152/ajplegacy.1975.228.5.1589

[B30] Honig C. R. Connett R. J. Gayeski T. E. (1992). O_2_ transport and its interaction with metabolism; a systems view of aerobic capacity. Med. Sci. Sports Exerc. 24, 47–53 10.1249/00005768-199201000-00009 1548995

[B31] Hub J. S. de Groot B. L. (2006). Does CO_2_ permeate through aquaporin-1? Biophys. J. 91, 842–848 10.1529/biophysj.106.081406 16698771 PMC1563782

[B32] Hub J. S. Winkler F. K. Merrick M. de Groot B. L. (2010). Potentials of mean force and permeabilities for carbon dioxide, ammonia, and water flux across a Rhesus protein channel and lipid membranes. J. Am. Chem. Soc. 132, 13251–13263 10.1021/ja102133x 20815391

[B33] Itada N. Forster R. E. (1977). Carbonic anhydrase activity in intact red blood cells measured with ^18^O exchange. J. Biol. Chem. 252, 3881–3890 405387

[B34] Itel F. Al-Samir S. Öberg F. Chami M. Kumar M. Supuran C. T. (2012). CO_2_ permeability of cell membranes is regulated by membrane cholesterol and protein gas channels. FASEB J. 26, 5182–5191 10.1096/fj.12-209916 22964306

[B35] Ivanov I. I. Fedorov G. E. Gus'kova R. A. Ivanov K. I. Rubin A. B. (2004). Permeability of lipid membranes to dioxygen. Biochem. Biophys. Res. Commun. 322, 746–750 10.1016/j.bbrc.2004.07.187 15336527

[B36] Jacobs M. H. (1920a). To what extent are the physiological effects of carbon dioxide due to hydrogen ions? Am. J. Physiol. 51, 321–331

[B37] Jacobs M. H. (1920b). The production of intracellular acidity by neutral and alkaline solutions containing carbon dioxide. Am. J. Physiol. 53, 457–463

[B38] Jacobs M. H. (1922). The influence of ammonium salts on cell reaction. J. Gen. Physiol. 5, 181–188 10.1085/jgp.5.2.181 19871986 PMC2140567

[B39] Kawashiro T. Scheid P. (1976). Measurement of Krogh's diffusion constant of CO_2_ in respiring muscle at various CO_2_ levels: evidence for facilitated diffusion. Pflügers Arch. 362, 127–133 10.1007/BF00583638 944419

[B40] Kikeri D. Sun A. Zeidel M. L. Hebert S. C. (1989). Cell membranes impermeable to NH_3_. Nature 339, 478–480 10.1038/339478a0 2725680

[B41] Kleinzeller A. (1997). Ernest Overton's contribution to the cell membrane concept: a centennial appreciation. News Physiol. Sci. 12, 49–53

[B42] Kutchai H. (1975). Role of the red cell membrane in oxygen uptake. Respir. Physiol. 23, 121–132 10.1016/0034-5687(75)90076-6 1129546

[B43] Landau L. D. Lifschitz E. M. (1991). Lehrbuch der Theoretischen Physik. Band VI, Hydrodynamik. 5. Auflage. Berlin: Akademie-Verlag

[B44] Lawrence J. H. Loomis W. F. Tobias C. A. Turpin F. H. (1946). Preliminary observations on the narcotic effect of xenon with a review of values for solubilities of gases in water and oils. J. Physiol. 105, 197–204 16991720 PMC1393631

[B45] Matsuzaki T. Hata H. Ozawa H. Takata K. (2009). Immunohistochemical localization of the aquaporins AQP1, AQP3, AQP4, and AQP5 in the mouse respiratory system. Acta Histochem. Cytochem. 42, 159–169 10.1267/ahc.09023 20126569 PMC2808499

[B46] Meyer zu Düttingdorf H. Sallmann H. Glockenthör U. von Engelhardt W. Busche R. (1999). Isolation and lipid composition of apical and basolateral membranes of colonic segments of guinea pig. Anal. Biochem. 269, 45–53 10.1006/abio.1998.3075 10094774

[B47] Mills G. A. Urey H. C. (1940). The kinetics of isotopic exchange between carbon dioxide, bicarbonate ion, carbonate ion and water. J. Am. Chem. Soc. 62, 1019–1026 10.1021/ja01862a010

[B48] Missner A. Kügler P. Saparov S. M. Sommer K. Mathai J. C. Zeidel M. L. (2008). Carbon dioxide transport through membranes. J. Biol. Chem. 283, 25340–25347 10.1074/jbc.M800096200 18617525 PMC2533081

[B49] Missner A. Pohl P. (2009). 110 years of the Meyer-Overton rule: predicting membrane permeability of gases and other small compounds. Chemphyschem 10, 1405–1414 10.1002/cphc.200900270 19514034 PMC3045804

[B50] Möller M. Botti H. Batthyany C. Rubbo H. Radi R. Denicola A. (2005). Direct measurement of nitric oxide and oxygen partitioning into liposomes and low density lipoprotein. J. Biol. Chem. 280, 8850–8854 10.1074/jbc.M413699200 15632138

[B51] Musa-Aziz R. Chen L. M. Pelletier M. F. Boron W. F. (2009). Relative CO_2_/NH_3_ selectivities of AQP1, AQP4, AQP5, AmtB, and RhAG. Proc. Natl. Acad. Sci. U.S.A. 106, 5406–5411 10.1073/pnas.0813231106 19273840 PMC2664022

[B52] Nakhoul N. L. Davis B. A. Romero M. F. Boron W. F. (1998). Effect of expressing the water channel aquaporin-1 on the CO_2_ permeability of *Xenopus* oocytes. Am. J. Physiol. 274, C543–C548 9486145 10.1152/ajpcell.1998.274.2.C543

[B53] Overton E. (1895). Über die osmotischen Eigenschaften der lebenden Pflanzen- und Tierzelle. Vierteljahrsschr. Naturforsch. Ges. Zürich 40, 159–201

[B54] Overton E. (1896). Über die osmotischen Eigenschaften der Zelle in ihrer Bedeutung für die Toxikologie und Pharmakologie. Vierteljahrsschr. Naturforsch. Ges. Zürich 41, 383–406

[B55] Overton E. (1899). Über die allgemeinen osmotischen Eigenschaften der Zelle, ihre vermutlichen Ursachen und ihre Bedeutung für die Physiologie. Vierteljahrssch. Naturforsch. Ges. Zürich 44, 88–114

[B56] Overton E. (1901). Studien über die Narkose. Jena: Gustav Fischer Verlag

[B57] Pfaller W. Gstraunthaler G. Kersting U. Oberleithner H. (1989). Carbonic anhydrase activity in Madin Darby canine kidney cells. Evidence for intercalated cell properties. Ren. Physiol. Biochem. 12, 328–337 2516353 10.1159/000173210

[B58] Prasad G. V. Coury L. A. Finn F. Zeidel M. L. (1998). Reconstituted aquaporin 1 water channels transport CO_2_ across membranes. J. Biol. Chem. 273, 33123–33126 10.1074/jbc.273.50.33123 9837877

[B59] Ripoche P. Bertrand O. Gane P. Birkenmeier C. Colin Y. Cartron J. P. (2004). Human Rhesus-associated glycoprotein mediates facilitated transport of NH3 into red blood cells. Proc. Natl. Acad. Sci. U.S.A. 101, 17222–17227 10.1073/pnas.0403704101 15572441 PMC535366

[B60] Roughton F. J. Forster R. E. (1957). Relative importance of diffusion and chemical reaction rates in determining rate of exchange of gases in the human lung, with special reference to true diffusing capacity of pulmonary membrane and volume of blood in the lung capillaries. J. Appl. Physiol. 11, 290–302 13475180 10.1152/jappl.1957.11.2.290

[B61] Roughton F. J. W. (1959). Diffusion and simultaneous reaction velocity in hemoglobin solutions and red cell suspensions. Progr. Biophys. Chem. 9, 55–104

[B62] Sakai H. Sato A. Masuda K. Takeoka S. Tsuchida E. (2008). Encapsulation of concentrated hemoglobin solution in phospholipid vesicles retards the reaction with NO, but not CO, by intracellular diffusion barrier. J. Biol. Chem. 283, 1508–1517 10.1074/jbc.M707660200 18003613

[B63] Schuster K. D. (1987). Diffusion limitation and limitation by chemical reactions during alveolar-capillary transfer of oxygen-labeled CO_2._ Respir. Physiol. 67, 13–22 10.1016/0034-5687(87)90003-X 3103184

[B64] Simon S. A. Gutknecht J. (1980). Solubility of carbon dioxide in lipid bilayer membranes and organic solvents. Biochim. Biophys. Acta 596, 352–358 10.1016/0005-2736(80)90122-4 6767496

[B65] Snedden W. Ledez K. Manson H. J. (1996). A new method for the measurement of gas solubility. J. Appl. Physiol. 80, 1371–1378 8926269 10.1152/jappl.1996.80.4.1371

[B66] Somersalo E. Occhipinti R. Boron W. F. Calvetti D. (2012). A reaction-diffusion model of CO2 influx into an oocyte. J. Theor. Biol. 309, 185–203 10.1016/j.jtbi.2012.06.016 22728674 PMC3471386

[B67] Subczynski W. K. Hopwood L. E. Hyde J. S. (1992). Is the mammalian cell plasma membrane a barrier to oxygen transport? J. Gen. Physiol. 100, 69–87 10.1085/jgp.100.1.69 1324973 PMC2229127

[B68] Swenson E. R. Deem S. Kerr M. E. Bidani A. (2002). Inhibition of aquaporin-mediated CO_2_ diffusion and voltage-gated H^+^ channels by zinc does not alter rabbit lung CO_2_ and NO excretion. Clin. Sci. (Lond). 103, 567–575 12444909 10.1042/cs1030567

[B69] Thews G. (1960). Ein Verfahren zur Bestimmung des O_2_-Diffusionskoeffizienten, der O_2_-Leitfähigkeit und des O_2_-Löslichkeitskoeffizienten im Gehirngewebe. Pflügers Arch. 271, 227–244 10.1007/BF00363006

[B70] Uehlein N. Lovisolo C. Siefritz F. Kaldenhoff R. (2003). The tobacco aquaporin NtAQP-1 is a membrane CO_2_ pore with physiological functions. Nature 425, 734–737 10.1038/nature02027 14520414

[B71] Uehlein N. Otto B. Eilingsfeld A. Itel F. Meier W. Kaldenhoff R. (2012). Gas-tight triblock-copolymer membranes are converted to CO_2_ permeable by insertion of plant aquaporins. Sci Rep. 2:538 10.1038/srep00538 22844579 PMC3406340

[B72] Verkman A. S. (1998). Role of aquaporin water channels in kidney and lung. Am. J. Med. Sci. 316, 310–320 10.1097/00000441-199811000-00004 9822113

[B73] Voter W. A. Gayeski T. E. (1995). Determination of myoglobin saturation of frozen specimens using a reflecting cryospectrophotometer. Am. J. Physiol. 269, H1328–H1341 7485565 10.1152/ajpheart.1995.269.4.H1328

[B74] Waisbren S. J. Geibel J. P. Modlin I. M. Boron W. F. (1994). Unusual permeability properties of gastric gland cells. Nature 368, 332–335 10.1038/368332a0 8127367

[B75] Wang Y. Cohen J. Boron W. F. Schulten K. Tajkhorshid E. (2007). Exploring gas permeability of cellular membranes and membrane channels with molecular dynamics. J. Struct. Biol. 157, 534–544 10.1016/j.jsb.2006.11.008 17306562

[B76] Wang Y. Tajkhorshid E. (2010). Nitric oxide conduction by the brain aquaporin AQP4. Proteins 78, 661–670 10.1002/prot.22595 19842162 PMC2805761

[B77] Widomska J. Raguz M. Subczynski W. K. (2007). Oxygen permeability of the lipid bilayer membrane made of calf lens lipids. Biochim. Biophys. Acta 1768, 2635–2645 10.1016/j.bbamem.2007.06.018 17662231 PMC2093700

[B78] Wistrand P. J. (1981). The importance of carbonic anhydrase B and C for the unloading of CO2 by the human erythrocyte. Acta Physiol. Scand. 113, 417–426 10.1111/j.1748-1716.1981.tb06918.x 6814190

[B79] Wunder M. A. Böllert P. Gros G. (1998). Mathematical modelling of the role of intra- and extracellular activity of carbonic anhydrase and membrane permeabilities of HCO^−^_3_, H_2_O and CO_2_ in ^18^O exchange. Isotopes Environ. Health Stud. 34, 197–205 10.1080/10256019808036371

[B80] Wunder M. A. Gros G. (1998). ^18^O exchange in suspensions of red blood cells: determination of parameters of mass spectrometer inlet system. Isotopes Environ. Health Stud. 34, 303–310 10.1080/10256019808234064 9919683

[B81] Wunder M. Gros G. (1997). Influence of membrane permeabilities for CO_2_, H_2_O and HCO^−^_3_ on O-18 exchange in suspensions of red cells. Pflügers Arch. Eur. J. Physiol. 433 Suppl., P556

[B82] Yang B. Fukuda N. van Hoek A. Matthay M. A. Ma T. Verkman A. S. (2000). Carbon dioxide permeability of aquaporin-1 measured in erythrocytes and lung of aquaporin-1 null mice and in reconstituted proteoliposomes. J. Biol. Chem. 275, 2686–2692 10.1074/jbc.275.4.2686 10644730

[B83] Zacharia I. G. Deen W. M. (2005). Diffusivity and solubility of nitric oxide in water and saline. Ann. Biomed. Eng. 33, 214–222 10.1007/s10439-005-8980-9 15771275

[B84a] Zhou Y. Bouyer P. Boron W. F. (2006). Evidence that AQP1 is a functional CO_2_ channel in proximal tubules. FASEB J. 20:A1225 (Abstract).

[B84] Zocher F. Zeidel M. L. Missner A. Sun T. T. Zhou G. Liao Y. (2012). Uroplakins do not restrict CO_2_ transport through urothelium. J. Biol. Chem. 287, 11011–11017 10.1074/jbc.M112.339283 22315218 PMC3322830

